# Thermo-Sensitive Alternative Splicing of *FLOWERING LOCUS M* Is Modulated by Cyclin-Dependent Kinase G2

**DOI:** 10.3389/fpls.2019.01680

**Published:** 2020-01-22

**Authors:** Candida Nibau, Marçal Gallemí, Despoina Dadarou, John H. Doonan, Nicola Cavallari

**Affiliations:** ^1^ Institute of Biological, Environmental, and Rural Sciences, Aberystwyth University, Aberystwyth, United Kingdom; ^2^ Institute of Science and Technology Austria, Klosterneuburg, Austria; ^3^ Max F. Perutz Laboratories, Medical University of Vienna, Vienna, Austria

**Keywords:** alternative splicing, cyclin-dependent kinase, temperature, FLOWERING LOCUS M, flowering time, *Arabidopsis thaliana*

## Abstract

The ability to sense environmental temperature and to coordinate growth and development accordingly, is critical to the reproductive success of plants. Flowering time is regulated at the level of gene expression by a complex network of factors that integrate environmental and developmental cues. One of the main players, involved in modulating flowering time in response to changes in ambient temperature is FLOWERING LOCUS M (FLM). *FLM* transcripts can undergo extensive alternative splicing producing multiple variants, of which *FLM-β* and *FLM-δ* are the most representative. While *FLM-β* codes for the flowering repressor FLM protein, translation of *FLM-δ* has the opposite effect on flowering. Here we show that the cyclin-dependent kinase G2 (CDKG2), together with its cognate cyclin, CYCLYN L1 (CYCL1) affects the alternative splicing of *FLM*, balancing the levels of *FLM-β* and *FLM-δ* across the ambient temperature range. In the absence of the CDKG2/CYCL1 complex, *FLM-β* expression is reduced while *FLM-δ* is increased in a temperature dependent manner and these changes are associated with an early flowering phenotype in the *cdkg2* mutant lines. In addition, we found that transcript variants retaining the full *FLM* intron 1 are sequestered in the cell nucleus. Strikingly, *FLM* intron 1 splicing is also regulated by CDKG2/CYCL1. Our results provide evidence that temperature and CDKs regulate the alternative splicing of *FLM*, contributing to flowering time definition.

## Introduction

Reproductive success in a constantly changing environment is a major challenge for all kingdoms of life and often involves adjusting behavior or development according to prevailing conditions. Higher plants, for example, can maximize their reproductive chances by timing their flowering to suit geographic location and seasonal weather patterns (Amasino, 2010).

While light is considered a master input in the transition to flowering (Liu, 2001; Weller et al., 2001; Searle and Coupland, 2004; Mattson and Erwin, 2005), temperature is also critical and can promote or delay flowering according to the species (Balasubramanian et al., 2006; Nakano et al., 2013; Romera-Branchat et al., 2014). Moreover, heat pulses can have mixed effects (Bouché et al., 2015) while prolonged cold exposure (a process called vernalization) has been long known to accelerate flowering in many species adapted to post-winter flowering (Chouard, 1960).

The switch from the vegetative to reproductive phase is coordinated by a large number of genes scattered across several pathways but a few *loci* can have a wide effect (Srikanth and Schmid, 2011; Strange et al., 2011; Song et al., 2013; Susila et al., 2018). *FLOWERING LOCUS C* (*FLC*), for example, has a major role in vernalization, ensuring that flowering does not occur until after winter, and variation at this *locus* has been implicated in determining fitness across geographic locations and plant lineages (Nordborg and Bergelson, 1999; Caicedo et al., 2004; Reeves et al., 2007). In this case, transition to flowering demands antisense-mediated chromatin silencing at the *FLC* locus and involves a complex regulatory array composed of *FLC* activators, like FRIGIDA, as well as repressors, the autonomous pathway, and cold (Whittaker and Dean, 2017).

Many *Arabidopsis* ecotypes require several weeks of vernalization to accelerate flowering (Lempe et al., 2005; Shindo et al., 2006), while commonly used accessions flower without prior exposure to cold (Johanson et al., 2000). Consequently, species-specific mechanisms regulate flowering time at ambient temperature and few have been reported at a detailed molecular level (Samach and Wigge, 2005; Lee et al., 2008; Wigge, 2013).

Unlike mammals (Vriens et al., 2014), plants lack a clear class of thermoreceptors but phytochrome B (phyB) and phototropins have been shown to fulfill dual roles as both thermo- and light-sensors (Jung et al., 2016; Legris et al., 2016; Fujii et al., 2017). In addition, many of the factors involved in temperature sensitive decisions in plants are still unknown and, therefore, our understanding of the molecular correlations in the temperature sensing/response pathways remains sketchy.

Increasing evidence points to a role of messenger RNA (mRNA) splicing as an endogenous “molecular thermometer” in temperature adaptations (Capovilla et al., 2015). Splicing is the removal of intronic (mostly non-coding) sequences from a primary transcript (pre-mRNA) to form the mature messenger RNA (mRNA) by a multi-megadalton complex called the spliceosome (Will and Luhrmann, 2011; Lee and Rio, 2015). Combinatorial recognition and usage of distinct nucleotide sequences (splice sites) on the pre-mRNA can lead to the assembly of multiple different transcripts in a process called alternative splicing (AS).

In higher plants, AS affects 60–70% of intron-containing genes (Chamala et al., 2015; Zhang et al., 2015). Hence, AS is a transcriptome-wide mechanism that has the potential to profoundly affect the level of gene expression in response to environmental stimuli. Indeed, generation of nonproductive transcript isoforms, targeted for non-sense-mediated mRNA decay (NMD) (Kalyna et al., 2012), or translation of protein variants with altered amino acid sequence and function can quickly modify the cell proteome (Marquez et al., 2015).

Several reports exploring different temperature ranges and environments have shown that AS plays a critical role in response to extreme (Mastrangelo et al., 2012; Leviatan et al., 2013; Staiger and Brown, 2013; Hartmann et al., 2016; Klepikova et al., 2016; Calixto et al., 2018; Laloum et al., 2018) as well as to very small variations in ambient temperature (Streitner et al., 2013; Capovilla et al., 2015; Pajoro et al., 2017; Verhage et al., 2017; Capovilla et al., 2018; James et al., 2018).

In *Arabidopsis*, ambient temperature modulation of flowering time involves the FLC-related MADS-box transcription factor FLOWERING LOCUS M (FLM, MAF1). Loss-of-function mutations in FLM reduce the temperature dependency of flowering suggesting its role as a repressor (Scortecci et al., 2001; Werner et al., 2005). Indeed, FLM modulates flowering time over a wide temperature range (from 5 to 23°C) and can bind, like FLC (Lee et al., 2007), to the SHORT VEGETATIVE PHASE protein (SVP), to form a potent FLM-SVP repressor complex (Lee et al., 2013; Posé et al., 2013). Besides FLM, flowering time is also regulated by the other FLC-clade proteins, MAF2–MAF5 (Ratcliffe, 2003; Li et al., 2008; Gu et al., 2013; Lee et al., 2013; Airoldi et al., 2015; Theißen et al., 2018).

Temperature information on *FLM* gene expression is integrated at the post-transcriptional level by the interplay of AS events leading to the production of several *FLM* mRNA forms (Capovilla et al., 2017). In the reference accession, Columbia-0 (Col-0), two of these variants, *FLM-β* and *FLM-δ*, are the predominant transcripts, which result from the alternative usage of the mutually exclusive exons 2 (*FLM-β*) and 3 (*FLM-δ*) (Lee et al., 2013; Posé et al., 2013).

The resulting proteins FLM-β and FLM-δ have been implicated in repressing or promoting flowering respectively. In particular, FLM-β was found to bind both SVP and to promoter regions of regulated target genes (Posé et al., 2013). Hence, FLM-β has been recognized as the real protagonist in the temperature dependent repressor complex while the function of FLM-δ as flowering promoter has been much debated (Capovilla et al., 2017). The effect of other FLM isoforms in flowering is also poorly understood.

Recently, specific splicing factors have been reported to modulate flowering time by affecting the balance between *FLM-β* and *FLM-δ*, like the U2 auxiliary factors ATU2AF65A and ATU2AF65B, the glycine rich proteins ATGRP7 and ATGRP8 and Splicing Factor 1 ATSF1 (Lee et al., 2017; Park et al., 2019; Steffen et al., 2019).

Cyclin-dependent kinases (CDKs), an evolutionarily conserved group of serine/threonine kinases initially implicated in cell cycle control (Strausfeld et al., 1996; Rane et al., 1999; Sherr and Roberts, 1999), are involved in pre-mRNA processing through interaction with the spliceosome components (Ko et al., 2001; Hu et al., 2003; Loyer et al., 2005; Even et al., 2006; Cheng et al., 2012). In plants, CDKs regulate a myriad of developmental processes including flowering time. CDKC, for instance is part of the positive transcription elongation factor b (P-TEFb) that phosphorylates the C-terminal domain of RNA polymerase II (PolII) (Cui et al., 2007), modulates the localization of spliceosome components (Kitsios et al., 2008) and can regulates flowering time through promoting expression of an *FLC* antisense transcript called *COOLAIR* (Wang et al., 2014). The CDKG group is the most closely related to mammalian CDKs (Menges et al., 2005; Umeda, 2005) that are involved in mRNA processing (Bartkowiak et al., 2010; Chen et al., 2006; Loyer et al., 2005; Even et al., 2006) and have been also shown to regulate splicing (Huang et al., 2013; Cavallari et al., 2018), meiosis (Zheng et al., 2014) and flowering responses (Ma et al., 2015).

In *Arabidopsis*, two closely related genes, *CDKG1* and *CDKG2*, encode for the catalytic subunit of the kinase, which physically interacts with the regulatory subunit, CYCLIN L1 (CYCL1) (Van Leene et al., 2010). CDKG1, CDKG2, and CYCL1 have a role in mRNA splicing (Xu et al., 2012; Huang et al., 2013) forming part of an ambient temperature responsive AS cascade targeting genes involved in splicing (Cavallari et al., 2018). Moreover, CDKG1 is required for chromosome pairing and recombination at high ambient temperature (Zheng et al., 2014) while CDKG2 was reported as a negative regulator of flowering (Ma et al., 2015) although the molecular pathway involved was not identified.

Here, we show that the early flowering phenotype in *cdkg2-1* as well as in the double *cdkg2-1;cycL1-1* mutant lines is maintained across the ambient temperature range and under different light conditions (both long and short day). Early flowering is associated with impaired AS of *FLM* transcripts as mutants in both the kinase and cyclin genes showed differential integration of temperature cues into *FLM* mRNA. Specifically, CDKG2 and CYCL1, but not CDKG1, are required for balancing *FLM-β* and *FLM-δ* levels across the ambient temperature range. Moreover, lack of CDKG2 and CYCL1 also affect the correct processing of the alternative introns 1 and 4 in *FLM* mRNAs. In addition, we report that mRNA variants retaining *FLM* intron 1 are sequestered in the cell nucleus.

Taken together our data provide evidence that the temperature pathways and the CDKG2/CYCL1 complex converge on the regulation of *FLM* AS to fine tune the flowering process.

## Materials and Methods

### Plant Materials and Growth Conditions

The wild type Columbia (Col-0) and mutant stocks *cdkg1-1 (*SALK_075762), *cdkg2-1* (SALK_012428), and *cycL1-1* (SAIL_285_G10) used in this study were obtained from the Nottingham Arabidopsis Stock Centre and have previously been described (Zheng et al., 2014; Ma et al., 2015; Cavallari et al., 2018).

For analysis of the splicing events, plants were grown in Petri-plates containing plant medium (0.5x MS salts and vitamins, pH 5.8, 0.7% plant agar) for 2 weeks at 23°C under either long day (LD) conditions (16 h light, 8 h dark) or short day (SD) conditions (8 h light, 16 h dark). Plants in LD or SD were then transferred to 15, 23, or 27°C for 2 days and collected for mRNA isolation. For the experiments listed above, Philips GreenPower LED production modules were used to provide a combination of red (660 nm)/far red (720 nm)/blue (455 nm), light with a photon density of about 140 µmolm^−2^s^−1^ +/−20%.

For the flowering experiments, seeds were sown in pots containing soil mix (80% Levington F2 and 20% sand) and placed at 15, 23, or 27°C either in LD (16 h light, 8 h dark) or SD (8 h light, 16 h dark). The light was provided by Sylvania 840 lamps and the light intensity 150 µmolm^−2^s^−1^ for LD and 250 µmolm^−2^s^−1^ for SD. Flowering was scored by counting the number of rosette leaves at bolting for each genotype.

### Ribonucleic Acid Extraction, Real Time, and Quantitative Polymerase Chain Reaction

Total RNA (3–5 seedlings per sample) was extracted from whole rosettes using the RNeasy Plant Mini kit (Qiagen). One microgram of total mRNA was used to generate cDNA using iScript™ cDNA Synthesis Kit (Bio-Rad). The primers used for the analysis of the AS of the different genes are listed in **Supplementary Table 2**. Three hundred eighty-four-well plates (Roche) were loaded using a JANUS Automated Workstation (PerkinElmer) with a 5 µl reaction containing 2.5 µl Luna^®^ Universal qPCR Master Mix (New England Biolabs). Quantitative PCRs (qPCRs) were performed using the LightCycler 480 (Roche). Samples (n≥3) were measured in technical triplicates and expression of *PP2AA3* (AT1G13320) was used as a reference (Czechowski, 2005). Data were analyzed using the LightCycler® 480 Software (Roche).

### Construct Generation and Plant Transformation

For transient expression in *Nicotiana benthamiana* leaves, the *CDKG2-GFP* (Cavallari et al., 2018) and *RSp34-RFP* (Lorković et al., 2004) cassettes were cloned into the pEAQ-HT-DEST2 vector (Sainsbury et al., 2009) and transformed into *Agrobacterium tumefaciens* strain LBA4404. Leaf infiltration was performed as described (Sainsbury et al., 2009). After 5 days, leaves were harvested for confocal imaging using a Leica TCS SP5 II confocal laser scanning microscope (CLSM) controlled by Leica LAS-AF software.

### Protoplast Isolation and Subsequent Cell Fractionation

Mesophyll protoplasts were isolated from 3-week-old Col-0 plants as described by Wu et al. (Wu et al., 2009). Subsequent cell fractions were prepared as described by Goehring et al. (Gohring et al., 2014) with slight modifications. Briefly, 2×10^6^ *Arabidopsis thaliana* mesophyll protoplasts were resuspended in 1 ml NIB lysis buffer [10 mM 2-(N-morpholino) ethanesulfonic acid-potassium hydroxide pH 5.5, 200 mM sucrose, 2.5 mM ethylenediaminetetraacetic acid, 2.5 mM dithiothreitol, 0.1 mM spermine, 10 mM NaCl, 0.2% Triton X-100, 1 U/µl RNasin (Promega)] and lysed using a 25 G gauge needle (6 to 10 passages). Complete lysis was confirmed by light microscopy. For the total fraction, 100 µl of lysed cells were immediately resuspended in 1 ml TRIzol (Ambion) and kept on ice until the remaining fractions were processed. The lysate was pelleted for 10 min at 500 g and 1 ml of supernatant, which represents the cytoplasmic fraction, was removed, and centrifuged for another 15 min at 10,000 g. Eight hundred µl of supernatant was resuspended in 8 ml TRIzol and the pellet, which represents the nuclear fraction, resuspended in 4 ml NRBT (20 mM Tris-HCl pH 7.5, 25% glycerol, 2.5 mM MgCl2, 0.2% Triton X-100), centrifuged at 500 g for 10 min and washed three times. After washing, the nuclear pellet was resuspended in 500 µl NRB2 (20 mM Tris-HCl pH 7.5, 250 mM sucrose, 10 mM MgCl2, 0.5% Triton X-100, 5 mM β-mercaptoethanol) and carefully overlaid on top of 500 µl NRB3 (20 mM Tris-HCl pH 7.5, 1.7 M sucrose, 10 mM MgCl2, 0.5% Triton X-100, 5 mM β-mercaptoethanol) and centrifuged at 16,000 g for 45 min. Finally, the nuclear pellet was resuspended in 1 ml TRIzol and RNA was isolated following the manufacturer’s instructions. Samples for protein analysis [total (whole protoplasts), cytoplasmic, and nuclear] were also kept.

### Protein Extraction and Western Blotting

Protein samples from the fractionation experiments (total, cytoplasmic, and nuclear) were resuspended in sample loading buffer and heated up at 65°C for 10 min before loading in a 10–20% polyacrylamide gradient gel (Bio-Rad) and transferred to polyvinylidene fluoride membranes. Membranes were probed with anti-H3 antibody (Abcam 1791) or anti-alcohol dehydrogenase (Agrisera AS10 685) at a dilution of 1:5,000 and the secondary antibody used was goat anti-rabbit immunoglobulin G coupled to unmodified horseradish peroxidase (Sigma) at a 1:10,000 dilution. Detection was done using the ECL Western Blotting Detection Reagent (Amersham) and signal detected with Image Quant LAS4000 (GE).

### Statistical Analysis

Statistical analyses were performed using PRISM 8 (GraphPad Software) or Excel (Microsoft Office, Microsoft). P-values were calculated using an unpaired, two-tailed Student’s t-test (***p < 0.001; **p < 0.01; *p < 0.05; ns, not significant). Unless otherwise indicated in the figure legend, data represent mean ± standard deviation.

## Results

### CKDG2 Regulates the Alternative Splicing of the Flowering Regulator *FLM*

**Figure 1:**
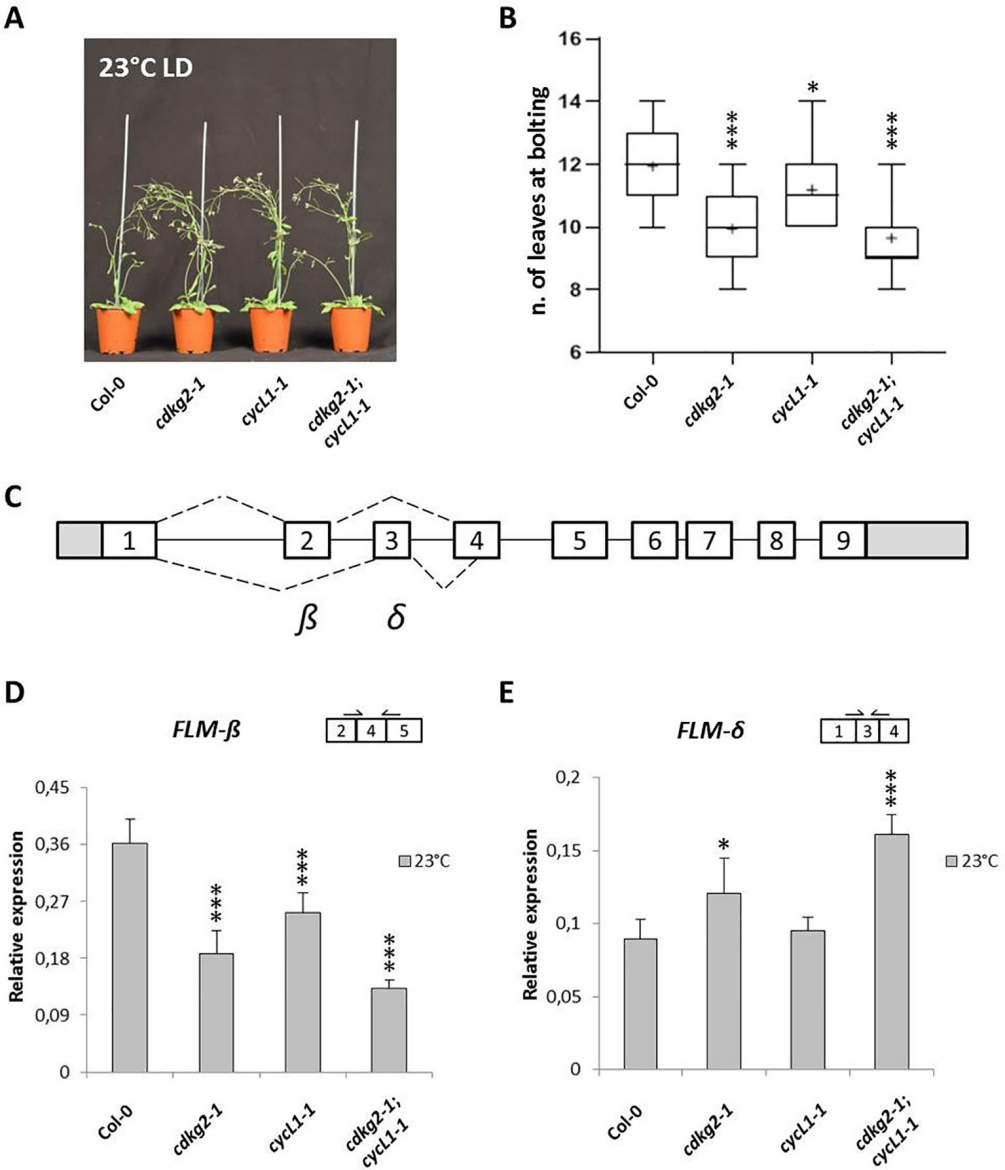
Lack of CDKG2 and CYCL1 is associated to early flowering and alters the alternative splicing of *FLM*. **(A)** Flowering phenotype of Col-0, *cdkg2-1*, *cycL1-1*, and *cdkg2-1;cycL1-1* mutants grown at 23°C under long day (LD) conditions. **(B)** Flowering time of the plants shown in **(A)** quantified by counting the number of rosette leaves present at bolting (n ≥ 30). Boxes represent 2^nd^ and 3^rd^ quartiles, bars minimum to maximum values, and crosses average of the groups. **(C)** Schematic representation of *FLM* locus and messenger RNA (mRNA) variants, including exons (boxes) and introns (lines). White boxes correspond to coding exons, gray boxes correspond to non‐coding exon sequences (UTRs). Dotted lines represent alternative splicing (AS) events. The major isoforms produced are also indicated (*β* and *δ*). **(D)** and **(E)** Relative expression levels of *FLM-β* **(D)** and *FLM-δ* **(E)** mRNA as quantified by real-time quantitative PCR in the different lines grown at 23°C under LD conditions (n ≥ 5). Student’s t-test comparing c*dkg2-1*, *cycL1-1*, or *cdkg2-1*;*cycL1-1* to Col-0, ***p < 0.001, and *p < 0.05.

It has been previously shown that the CDKG group of kinases and their cognate cyclin, CYCLIN L1 (CYCL1), are important regulators of temperature dependent AS in *Arabidopsis* (Huang et al., 2013; Cavallari et al., 2018). Moreover, plants lacking CDKG2 display an early flowering phenotype when grown at ambient temperature (Ma et al., 2015). This led us to hypothesize that the early flowering phenotype in *cdkg2-1* mutant lines could be maintained along the ambient temperature range as a result of defective AS in genes involved in the temperature transduction pathway. To test this possibility, we grew wild type, single *cdkg2-1* and *cycL1-1*, and the double *cdkg2-1*;*cycL1-1* mutant lines at 23°C under a LD light regime. Under these conditions, both the single *cdkg2-1* and *cycL1-1* and the double *cdkg2-1*;*cycL1-1* mutants flowered significantly earlier than the wild type (**Figures 1A, B**). On the contrary, no flowering phenotype was observed in the *cdkg1-1* mutant line (**Supplementary Figure 1A**).

Subsequently, we conducted a reverse transcriptase-polymerase chain reaction (RT-PCR) screen in cdkg mutant lines to test AS and expression levels of a small panel of genes including splicing factors, clock genes, and flowering regulators (**Supplementary Table 1**). Remarkably, among the investigated targets we found that in the single *cdkg2-1*, *cycL1-1* and in the double *cdkg2-1*;*cycL1-1* mutant lines, the processing of *FLM* (*MAF1*), a master regulator of the ambient temperature flowering pathway, was altered in terms of the relative levels of *FLM-β* and *FLM-δ* transcripts (**Supplementary Figure 1B**). In contrast the AS of *MAF2* (Airoldi et al., 2015), a close *FLM* paralogue was not affected in the different lines (**Supplementary Figure 1C**). In addition, no differences in the splicing of *FLM* or *MAF2* were observed in the single *cdkg1-1* mutant (**Supplementary Figures 1B, C**). The double *cdkg1-1;cdkg2-1* loss of function line could not be assessed as this genotype cannot be recovered and is assumed to be lethal (Zheng et al., 2014).

In order to investigate the changes in AS in more detail we quantified the levels of *FLM-β* and *FLM-δ* transcripts in the different mutants grown at 23°C by quantitative RT-PCR (RT-qPCR, see **Figure 1C** and **Supplemental Figure 2A** for *FLM* gene structure, AS events, and primer position). As observed by RT-PCR, lower levels of *FLM-β* (coding for the flowering repressor isoform) and increased expression of *FLM-δ* were observed in the single *cdkg2-1* and *cycL1-1* and in the double *cdkg2-1;cycL1-1* mutant lines (**Figures 1D, E**). Specifically, *FLM-β* expression was severely reduced in *cdkg2-1* (0.52 ± 0.10 fold) and in *cycL1-1* (0.70 ± 0.09 fold) and further impaired in the double *cdkg2-1*;*cycL1-1* (0.36 ± 0.03 fold) mutant lines in comparison to Col-0 (**Figure 1D**). The levels of *FLM-δ* were instead found significantly higher (up to 1.8 fold) in both *cdkg2-1* and *cdkg2-1*;*cycL1-1* mutants compared to wild type (**Figure 1E**) suggesting that CDKG2 together with CYCL1 maintains the balance between these two mutually exclusive isoforms. No significant change in *FLM-β* and *FLM-δ* expression were found between Col-0 and the *cdkg1-1* mutant lines (**Supplementary Figures 3A, B**).

Analysis of other flowering regulators involved in the temperature pathway showed that while there were no differences in the expression levels for *FLC* and the *TEMPRANILLO* genes (*TEM1* and *TEM2*; **Supplementary Figures 4A, B**), total *SVP* transcripts were reduced in all the mutant lines in comparison to Col-0 (**Supplementary Figure 4C**). This was mostly due to a reduction in the expression of one of two major *SVP* isoforms, *SVP2*, in mutant lines as determined by RT-PCR. Moreover, the lack of CDKG2 did not affect the AS of *FLM* regulatory genes like *ATU2AF65A* (Cavallari et al., 2018), *ATSF1*, or *ATGRP7* (**Supplementary Figure 4D**).

Consistent with its role in splicing, the CDKG2-GFP protein localizes to the nucleus of plant cells where it co-localizes with the spliceosome component RSp34-RFP (**Supplementary Figures 5A, B**).

### Altered *FLM* Splicing in *cdkg2* Mutants Is Associated With Early Flowering Across Different Temperatures

**Figure 2:**
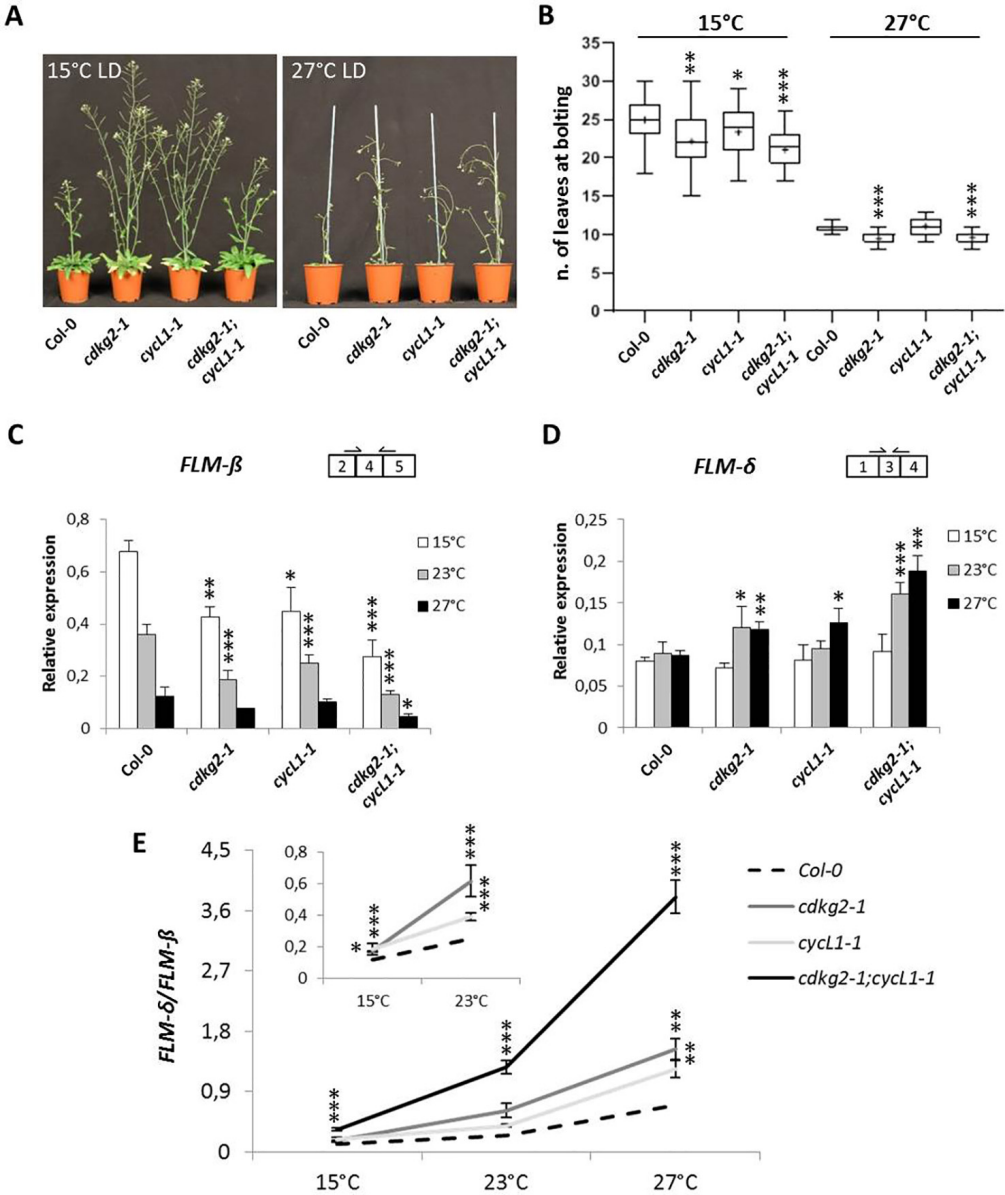
Lack of CDKG2 and CYCL1 unbalances the alternative splicing of *FLM* across the ambient temperature range. **(A)** Flowering phenotype of Col-0, *cdkg2-1*, *cycL1-1*, and *cdkg2-1;cycL1-1* mutants grown under long day (LD) conditions at 15 and at 27°C as indicated. **(B)** Flowering time of the plants shown in **(A)** quantified by counting the number of rosette leaves present at bolting (n ≥ 27 at 15°C and n ≥ 23 at 27°C). Boxes represent 2^nd^ and 3^rd^ quartiles, bars minimum to maximum values, and crosses average of the groups. **(C)** and **(D)** Relative expression levels of *FLM-β* **(C)** and *FLM-δ* **(D)** messenger RNA (mRNA) as quantified by real-time quantitative PCR in the different lines, grown at 15, 27, and 23°C for comparison under LD conditions as indicated (n ≥ 3). **(E)** Ratio of *FLM-δ*/*FLM-β* mRNA in *cdkg2-1, cycL1-1*, and *cdkg2-1;cycL1-1* in comparison to Col-0 at the respective temperature (LD, long day). In the inset, detail of Col-0, *cdkg2-1*, and *cycL1-1* for statistic display. Student’s t-test ***p < 0.001, **p < 0.01, and *p < 0.05.

We have previously shown that the CDKG group is actively involved in maintaining plant homeostasis along the ambient temperature range (Zheng et al., 2014; Cavallari et al., 2018). This led us to test the possibility that the lack of CDKG2 or of its co-factor CYCL1 may regulate flowering along the ambient temperature range. For this, plants were grown at both 15 and 27°C under LD conditions (LD, 16 h light/8 h dark). As observed at 23°C, the early flowering phenotype of the single *cdkg2-1* and double *cdkg2-1;cycL1-1* was maintained at the different temperatures tested (**Figures 2A, B**) albeit with some small differences. At 15°C the double mutant lines flowered slightly earlier than the single *cdkg2-1* and *cycL1-1* lines (**Figures 2A, B**). At 27°C, both the single *cdkg2-1* and the double mutant line were flowering significantly earlier than the wild type (**Figures 2A, B**) while no significant differences in flowering time were seen for the *cycL1-1* mutant.

To determine the effect of temperature on *FLM* splicing in the different mutant backgrounds, we quantified the levels of *FLM-β* and *FLM-δ* by RT-qPCR in 2-week old seedlings grown under LD conditions by shifting growth temperature from 23°C either to 15°C or to 27°C for 48 h before sampling.

In the wild type, *FLM-β* levels displayed temperature sensitivity as previously reported (Posé et al., 2013) with transcript levels raising at 15°C and decreasing at 27°C (**Figure 2C**) while *FLM-δ* expression remained relatively stable in wild type (**Figure 2D**). Strikingly, we found a more pronounced reduction in *FLM-β* levels along the temperature range in mutant lines and a significant increase in *FLM-δ* at 23 and 27°C (**Figures 2C, D**) in comparison to wild type. The detrimental effect of temperature increases on splicing in the mutant lines became more evident when the ratio between *FLM-β* and *FLM-δ (FLM-δ/FLM-β)* was calculated at each temperature point (**Figure 2E**). While in Col-0 the ratio increased with the temperature (5.9 ± 1.2 fold from 15 to 27°C) this increase was higher in the mutant lines (11.5 ± 0.7 fold in *cdkg2-1*;*cycL1-1*).

We also examined the relative levels of *SVP* expression in the various mutant backgrounds at different temperatures and found that the double *cdkg2-1;cycL1-1 mutant* had constitutively lower *SVP* expression than the wt control across the temperature range (**Supplementary Figure 6A**).

Although FLM is known to influence flowering particularly at lower temperatures (Lutz et al., 2015), the *cdkg2-1* and the *cdkg2-1;cycL1-1* double mutants flowered earlier than Col-0 also at 27°C suggesting the involvement of additional regulatory mechanisms. Expression of *FLC* was reported to have a strong impact on flowering time particularly at high temperatures (Balasubramanian et al., 2006). However, we observed no significant changes in *FLC* expression between Col-0 and the mutant lines at 27°C suggesting that the early flowering phenotype of the *cdkg2* mutants is not due to altered *FLC* expression (**Supplementary Figure 6B**).

### CDKG2/CYCL1 Has a Wide Effect on *FLM* Transcript Processing

**Figure 3:**
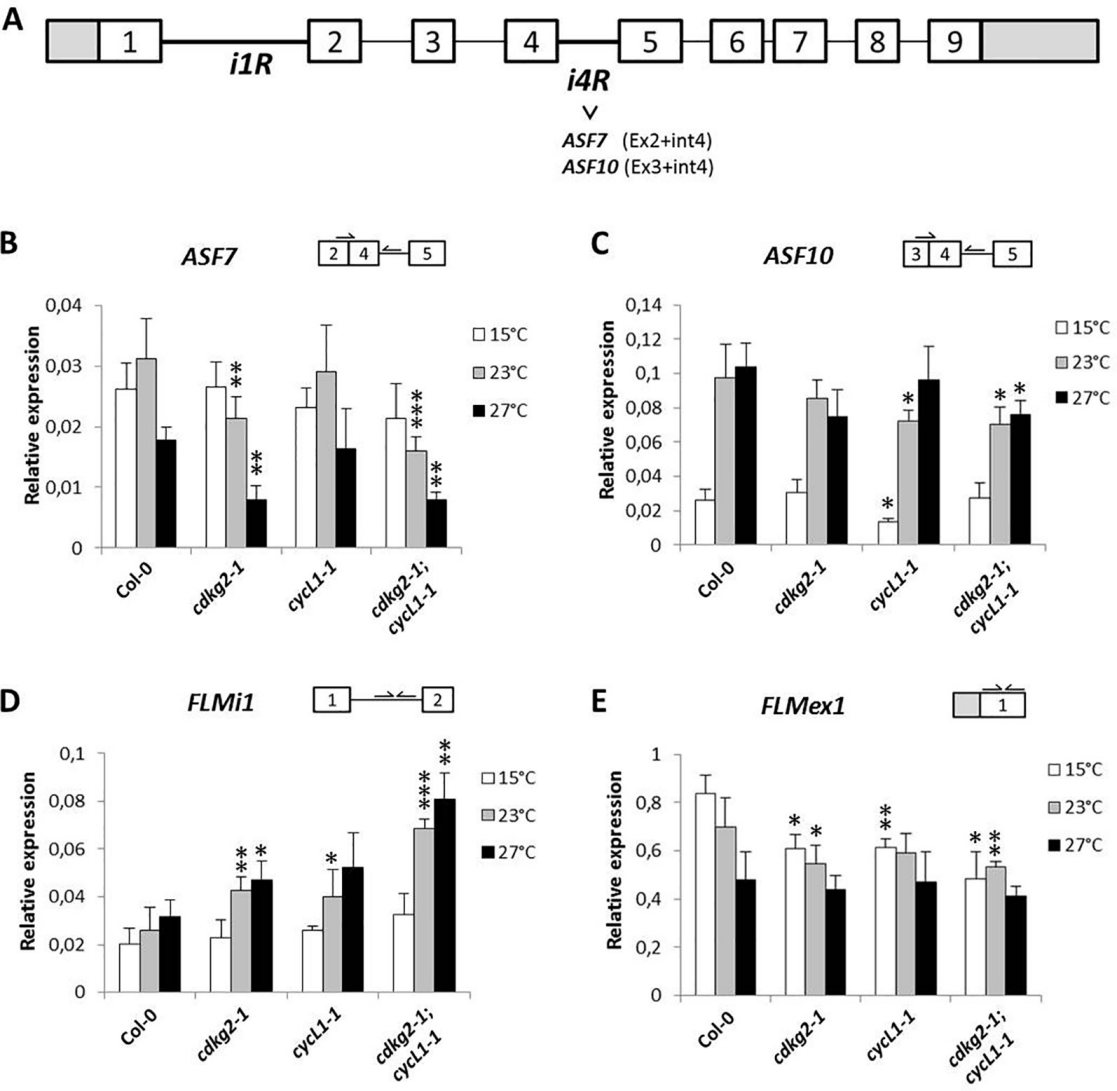
CDKG2 and CYCL1 also regulate the abundance of other *FLM* alternative splicing isoforms. **(A)** Schematic representation of *FLM* locus and messenger RNA (mRNA) variants, including exons (boxes) and introns (lines). White boxes correspond to coding exons, gray boxes correspond to non‐coding exon sequences (UTRs). iR, intron retention. **(B**–**E)** Relative expression levels of *ASF7* **(B)**, *ASF10* **(C)**, *FLMi1* **(D)**, and *FLMex1* **(E)** mRNA as quantified by real-time quantitative PCR in the different lines, grown at 15, 23, and 27°C under long day conditions as indicated. Student’s t-test comparing *cdkg2-1*, *cycL1-1*, or *cdkg2-1*;*cycL1-1* to Col-0 at the respective temperature, n ≥ 3, ***p < 0.001, **p < 0.01, and *p < 0.05.

In order to determine if other major splicing events in *FLM* were affected by the lack of CDKG2 and CYCL1, we analyzed expression of the mRNAs that retain intron 4, namely splicing variants *ASF7* or *ASF10* (Capovilla et al., 2017). Retention of the in frame intron 4, either in combination with exon 2 or 3, could translate for proteins with characteristics similar to FLM-β or FLM-δ respectively (see **Figure 3A** and **Supplementary Figure 2A** for splicing scheme). Remarkably, we found reduced levels of intron 4 retention for *ASF7* transcripts in the *cdkg2-1* and *cdkg2-1;cycL1-1* mutant lines at 23 and 27°C while *ASF10* was mildly but significantly affected in the single *cycL1-1* and in the double mutant albeit at different temperatures (**Figures 3B, C**).

Variations in *FLM* intron 1 sequence were previously shown to fine tune flowering time and to be involved in adaptation to temperature (Lutz et al., 2015; Lutz et al., 2017) and based on database annotations (Araport11) there are several potential intron 1 retention *FLM* mRNAs (AT1G77080.6, AT1G77080.7, AT1G77080.9, AT1G77080.10). These alternative *FLM* transcripts could thus affect *FLM* expression. We observed that *FLM* intron 1 retention (*FLMi1*) was not affected by temperature in Col-0 while single and double mutant lines showed remarkably higher retention levels at 23 and 27°C (up to 2.6 ± 0.34 fold, **Figure 3D**).

Taken together, these data suggested that the lack of CDKG2 and CYCL1 affected not only the balance between *FLM-β* and *FLM-δ* but also the processing of other *FLM* transcripts spanning from exon 1 to intron 4 along the ambient temperature range.

The observed differences in the relative *FLM* isoform abundance and how these may impact on the expression of *FLM*, prompted us to evaluate the total levels of *FLM* by measuring *FLM* exon 1 (*FLMex1*) containing transcripts by RT-qPCR. Total levels of *FLM* mRNA decreased along the temperature range in Col-0 and were further reduced in the mutant lines both at 15 and 23°C but not at 27°C (**Figure 3E**). These observations suggest that the lower *FLM* levels observed in the *cdkg2-1* and *cycl1-1* mutants may reflect intrinsic differences in *FLM* isoform stability, although we cannot completely exclude a concomitant reduction in transcription at specific temperatures.

### Lack of CDKG2 and CYCL1 Promotes Flowering and Alters *FLM* Alternative Splicing Independently of the Photoperiod

Since the photoperiod also has a strong effect on flowering time, we assessed the flowering phenotypes of the single and double *cdkg2-1* and *cycL1-1* mutants under SD conditions. For this we grew plants at 15, 23, and 27°C in SD (8 h light/16 h dark) which is considered a non-inductive condition for *Arabidopsis* (Balasubramanian and Weigel, 2006). We hypothesize if mainly the temperature pathway was affected then early flowering should be maintained independently of day length.

**Figure 4:**
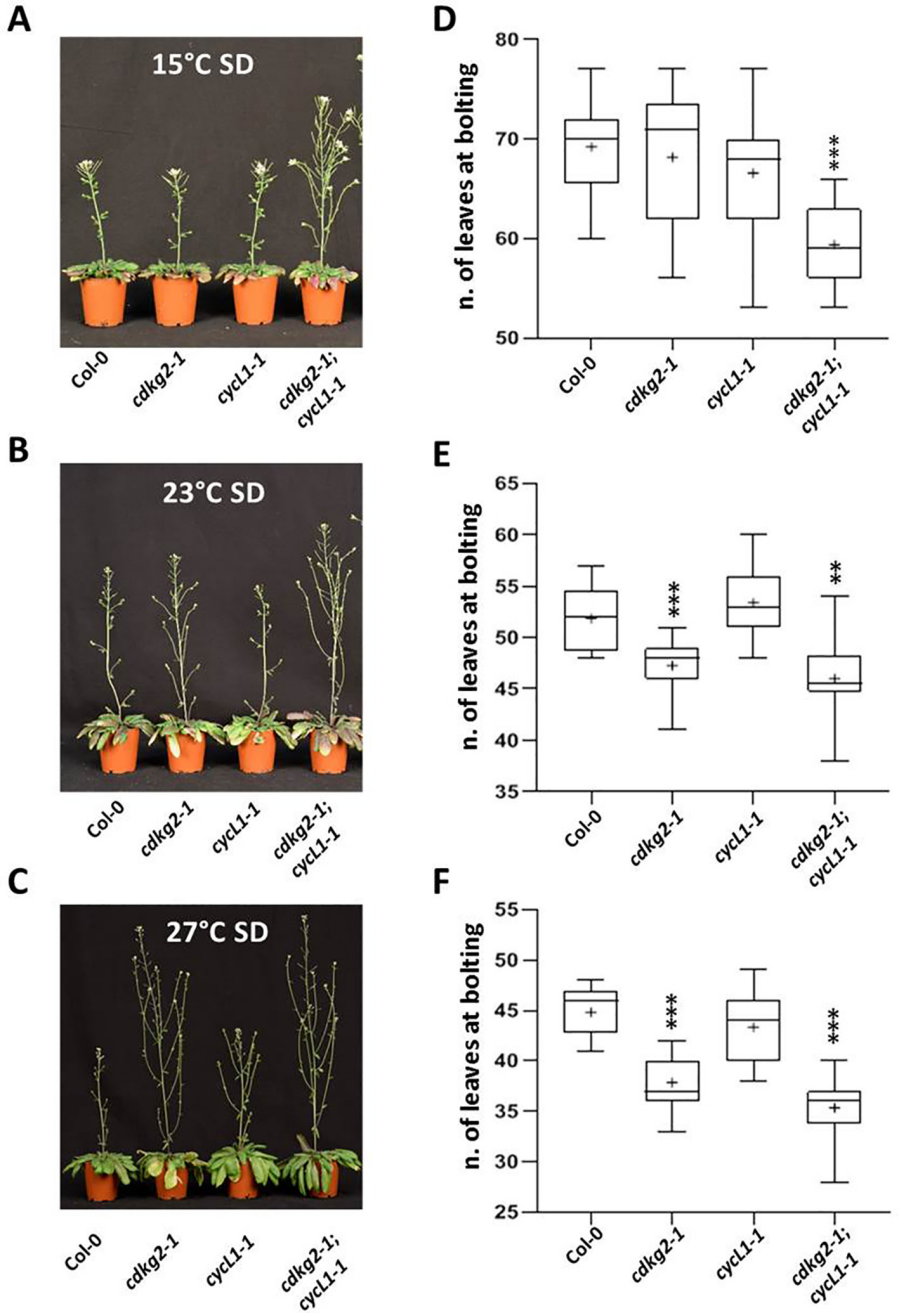
Lack of CDKG2/CYCL1 complex promotes flowering across the ambient temperature range in short day conditions. **(A**–**C)** Flowering phenotype of Col-0, *cdkg2-1*, *cycL1-1*, and *cdkg2-1;cycL1-1* mutants grown under SD conditions at 15°C **(A)**, at 23°C **(B)**, and at 27°C **(C)**. **(D**–**F)** Flowering time of the plants shown in **(A)**, **(B)**, and **(C)** quantified by counting the number of rosette leaves present at bolting (n ≥ 15, n ≥ 10, and n ≥ 14 respectively). Boxes represent 2^nd^ and 3^rd^ quartiles, bars minimum to maximum values, and crosses average of the groups. Student’s t-test comparing *cdkg2-1*, *cycL1-1*, or *cdkg2-1*;*cycL1-1* to Col-0 at the respective temperature, ***p < 0.001 and **p < 0.01.

The double *cdkg2-1;cycL1-1* mutant lines still flowered earlier than wild type plants in SD conditions at all temperatures while in *cdkg2-1* this effect was present at 23°C and at 27°C (**Figures 4A–F**).

**Figure 5:**
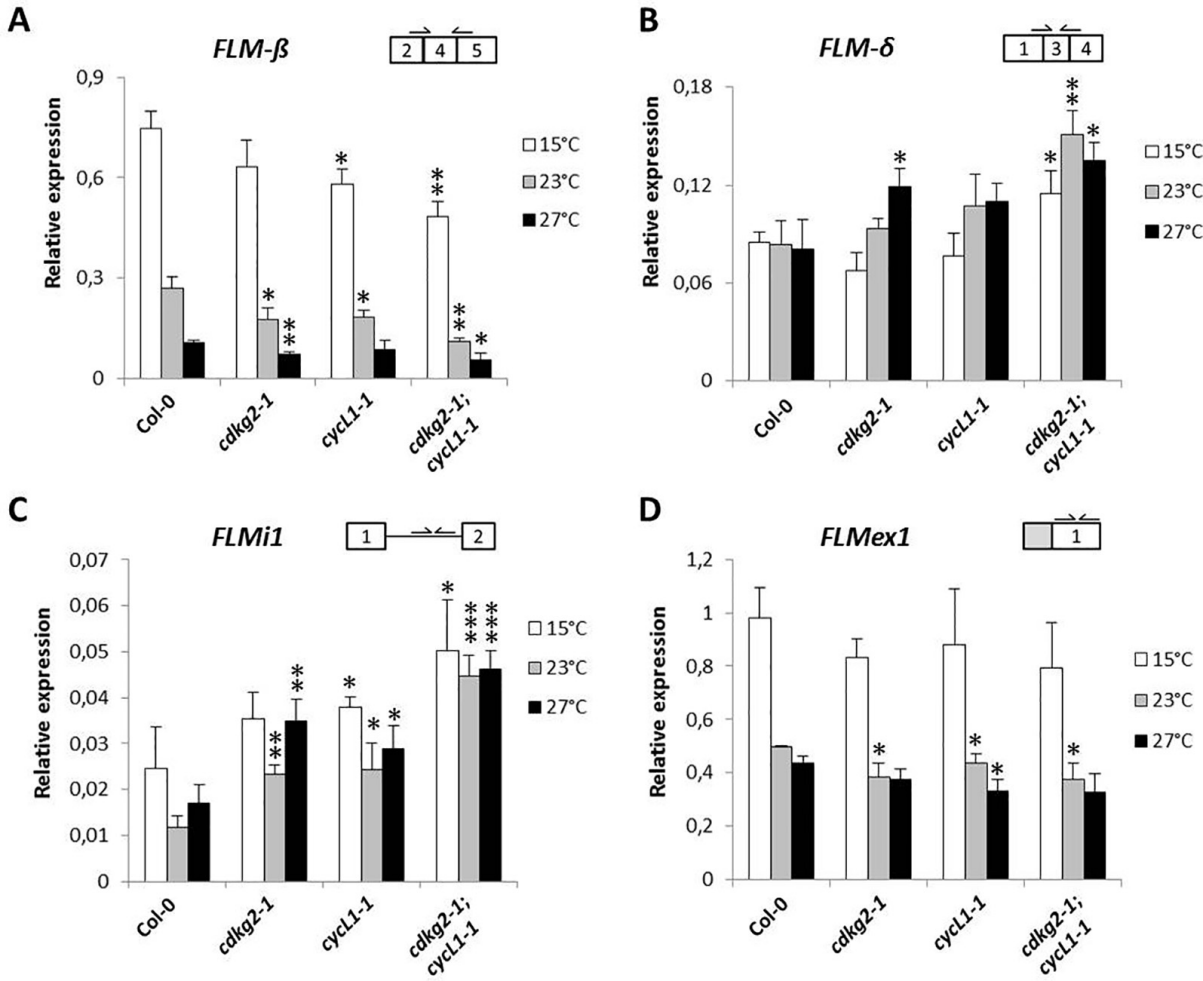
Lack of CDKG2 and CYCL1 unbalances the alternative splicing of *FLM* across the ambient temperature range in short day (SD) conditions. **(A**–**D)** Relative expression levels of *FLM-β* **(A)**, *FLM-δ* **(B)**, *FLMi1* **(C)**, and *FLMex1* **(D)** messenger RNA as quantified by real-time quantitative PCR in the different lines, Col-0, *cdkg2-1*, *cycL1-1*, *cdkg2-1;cycL1-1* grown at 15, 23, and 27°C under SD conditions (8 h light, 16 h dark) as indicated. Student’s t-test comparing *cdkg2-1*, *cycL1-1*, or *cdkg2-1*;*cycL1-1* to Col-0 at the respective temperature, n = 3, ***p < 0.001, **p < 0.01, and *p < 0.05.

As we observed under LD conditions, plants grown in SD showed small decreases in *FLM-β* with increased temperature (**Figure 5A**). Increases in *FLM-δ* transcripts were significant only in the double *cdkg2-1;cycL1-1* mutant (**Figure 5B**). In addition, *FLMi1* levels were also increased in mutant lines while *FLMex1* was lower mainly at 23°C (**Figures 5C, D**). Since, no differences in flowering were observed in SD conditions (23°C) and expression of *FLM-β* and *FLM-δ* was similar between Col-0 and the *cdkg1-1* mutant line (**Supplementary Figures 7A–C**), we decided not to further test this mutant in the present investigation.

The data profile obtained for *ASF7* and *ASF10* under SD conditions was comparable to that seen under LD conditions. Indeed, *ASF7* levels decreased with temperature and this effect was more accentuated in the mutants at higher temperatures. The effect on *ASF10* was less pronounced (**Supplementary Figures 8A, B**). Notably, the expression profile of *SVP* was not affected in the mutant lines in SD conditions (**Supplementary Figure 8C**).

Taken together, these results suggest that the temperature-dependent effect of the CDKG2/CYCL1 complex on the AS of *FLM* is independent of the photoperiod.

### FLMi1 Transcripts Accumulate in the Cell Nucleus

Intron retention events in plants can promote mRNA sequestration in the nucleus (Gohring et al., 2014) so that the affected mRNAs are unlikely to be translated into proteins in the cytoplasm. Hence, a possible consequence of the significant increase in *FLM* intron 1 containing transcripts in the mutant lines could be the increase of the nuclear *FLM* mRNA pool. This could represent an interesting, yet previously unknown, mechanism of *FLM* regulation based on CDKG2 activity and controlling *FLM* nuclear export.

The accumulation of intron 1 containing transcripts in the *cdkg2-1* and *cycL1-1* single and double mutants could be the consequence of either an increase in *FLM* pre-mRNA (unprocessed transcripts) or of a specific CDKG2 effect on intron 1 AS. To distinguish between these two possibilities, we amplified only processed messengers by RT-PCR by positioning the primers at the end of *FLM* intron 1 and at the exon 4/exon 5 junction (FLMi1e2F and FLMe5-4R; **Supplementary Table 2**). Interestingly, the transcripts we found had size corresponding to *FLMi1* mRNAs that contain both intron 2 and intron 3 (and relative exons) or only intron 2 (**Supplementary Figure 9A**). Moreover, we observed that these isoforms where more abundant in the double *cdkg2-1;cycL1-1* mutant than in Col-0, confirming that we see increased *FLM* intron 1 retention in the absence of CDKG2/CYCL1 (**Supplementary Figure 9A**).

**Figure 6:**
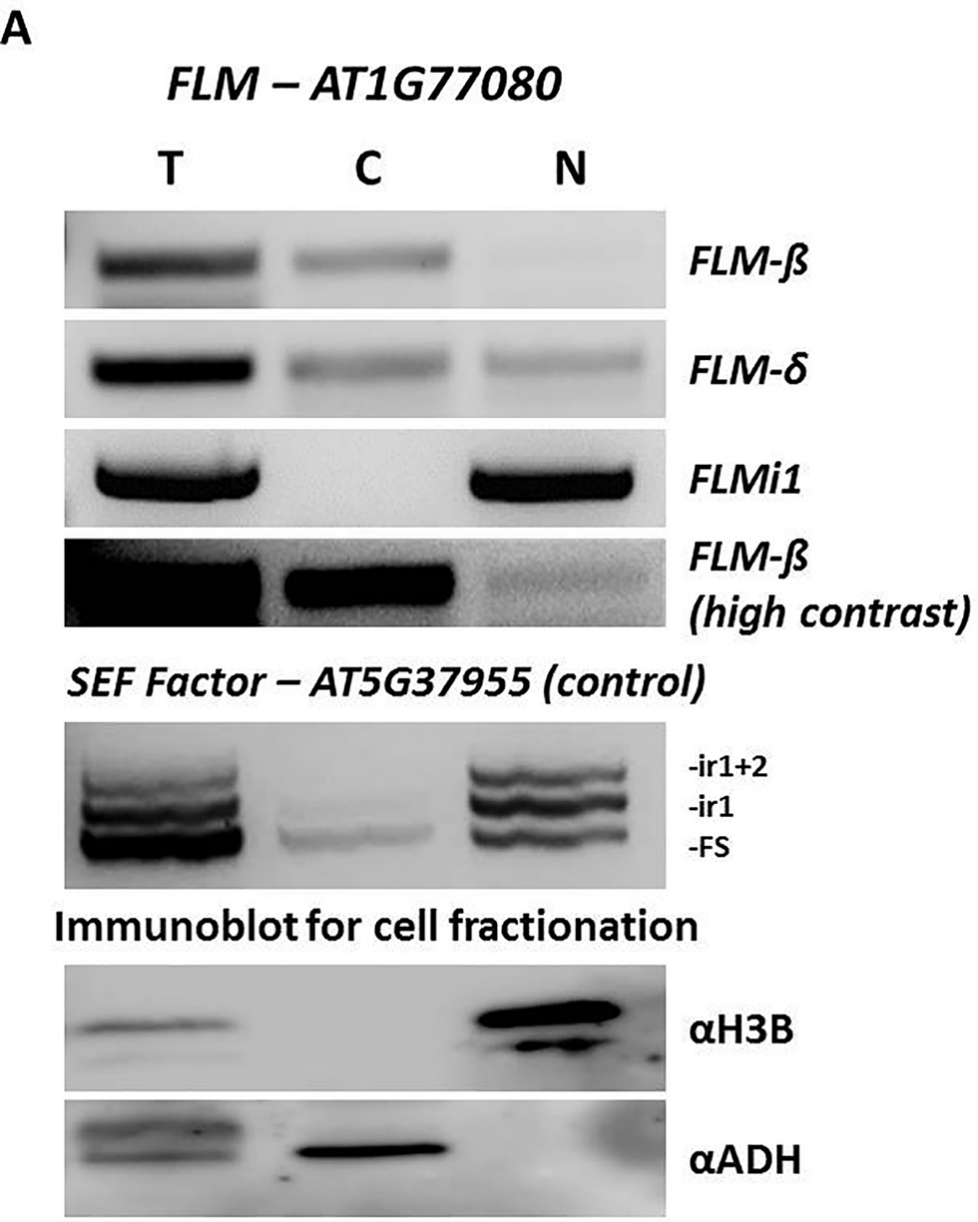
*FLM* intron 1 retention prevents nuclear export of *FLM* messenger RNA. **(A)** Expression analysis and sub-cellular localization of different FLM isoforms. Top panels, *FLM-β*, *FLM-δ*, and *FLMi1*, in fractionated cell extracts (T-total, C-cytoplasmic, N-nuclear) by RT-PCR. *FLM-β* (high contrast) show nuclear localization for this isoform. Middle panel, The SEF factor splice variants in fractionated cell extracts. The *SEF* factor was used as a fractionation control as only the mature form (FS) is exported to the cytoplasm. FS, fully spliced; ir1, intron 1 retention; ir1+2, retention of introns 1 and 2. Lower panels, polyacrylamide gel electrophoresis of protein extracts from the same cell fractionation. The cytoplasmic fraction is free of the nuclear protein histone 3B (H3B), and the nuclear fraction is free of the cytoplasmic alcohol dehydrogenase (ADH).

These findings prompted us to fractionate protoplast cell mRNA and assess sub-cellular localization of specific transcripts. Strikingly, nuclear and cytoplasmic fractions showed that while *FLM-β* and *FLM-δ* forms are present in the cytoplasm (as expected, being protein coding isoforms) *FLMi1* was retained in the nucleus (**Figure 6A**). The purity of the fractions was confirmed by RT-PCR for *SEF Factor* (AT5G37955) and by Western blot.

In summary, the data presented here show that the CDKG2/CYCL1 complex affects the temperature-dependent splicing of *FLM*. Finally, the nuclear retention of *FLM* intron1 containing transcripts could provide a new layer of *FLM* regulation across the temperature range.

## Discussion

The identification of key components in ambient temperature sensing/response in plants is crucial not least in times of global warming where increased temperature variation could produce ecological changes that will negatively impact on the present agricultural system (Wheeler and von Braun, 2013; Moore and Lobell, 2015; Jagadish et al., 2016). Hence, investigation and analysis of the molecular circuits involved in the temperature transduction pathways in plants is now of considerable importance.

While animals have developed specialized receptor classes for specific environmental variables (Terakita and Nagata, 2014; Vriens et al., 2014), the sensors so far identified in plants belong to diverse gene families and can have wider roles in both sensing and integrating environmental cues (Paik and Huq, 2019). The CDKG group of kinases, for example, has an important role in inherently temperature sensitive processes like meiosis and flowering (Zheng et al., 2014; Ma et al., 2015).

Recently we found that CDKGs can also integrate ambient temperature inputs by modulating an alternative mRNA splicing cascade (Cavallari et al., 2018) raising the question as to whether the role of CDKs in the aforementioned developmental processes could be acting through AS.

In the current report, we demonstrate that the CDKG2/CYCL1 modulates AS of the flowering regulator *FLM*, possibly providing an additional mechanism fine-tuning flowering time across the ambient temperature range.

*FLM* mRNA processing responds strongly to ambient temperature coding for some known (i.e., FLM-β and FLM-δ) as well as putative isoforms (i.e., ASF7 and ASF10) (Posé et al., 2013; Capovilla et al., 2017). While the repressive role of FLM-β in flowering time regulation is well accepted there is still debate about the function of FLM-δ. In addition, functional characterization of ASF7 and ASF10 proteins (with predictably similar functions as FLM-β and FLM-δ) is still missing. Indeed, *ASF7* and *ASF10* transcripts contain the in-frame *FLM* intron 4 which belongs to the exitron class (Marquez et al., 2015). Exitrons define a particular intron group associated with translation of alternative protein variants, suggesting that *ASF7* and *ASF10* might code for alternative proteins with different (and as yet unknown) functions.

Besides the strong temperature regulation of *FLM* AS, we found that the absence of CDKG2 and CYCL1 resulted in changes in the abundance of *FLM-β* and *FLM-δ* and, to a minor extent, of *ASF7* and *ASF10* across the temperature range (**Figures 2C, D** and **3B, C**) and under LD and SD conditions (**Figures 5A, B** and **Supplementary Figures 8A, B**). While temperature increases affects levels of the active floral repressor *FLM-β*, CDKG2 acted against the temperature signal to dampen the shift on the production of its non-repressive counterpart *FLM-δ*.

Moreover, CDKG2 and CYCL1 control the levels of *FLM* intron 1 retention and this new regulatory mechanism may influence the *FLM* intracellular mRNA trafficking (Reed, 2003). The nuclear retained *FLMi1* mRNAs could potentially be further processed, as was recently shown for the splicing factor *SR30* (Hartmann et al., 2018), and be stored or released from the cell nucleus in response to changing environmental conditions to promote or delay transition to flowering respectively. Indeed, the two *FLMi1* isoforms found by RT-PCR (**Supplementary Figure 6A**) retaining intron 2 and intron 3 may be spliced either into *FLM-β* or *FLM-δ* variants.

Hence, modulation of CDKG2 kinase activity is likely to impact on flowering time definition changing the AS of *FLM*, either by altering the ratio of *FLM-β* and *FLM-δ* as reported for other splicing factors (Lee et al., 2017; Park et al., 2019; Steffen et al., 2019) or by promoting retention of *FLM* intron 1. Indeed, the predicted increase in nuclear retention for *FLMi1* isoforms would provide a new additional, elegant, and rapid signaling module to adjust flowering time in response to changes in ambient temperature. Furthermore, the observation that the effect on AS in mutant lines was greater at higher temperatures (**Figure 2E**) suggests that CDKGs may contribute to temperature compensation during mRNA processing, a feature which is very important for other cellular mechanisms like the circadian clock (Avello et al., 2019). Consistent with this idea, *FLM-δ* and *FLMi1* expression became temperature dependent in *cdkg2-1* mutant lines, contrary to Col-0 where these isoforms were stably expressed (**Figures 2D** and **3D**).

Previously we showed that CDKG1 affected the splicing of *ATU2AF65A* (Cavallari et al., 2018) and recently, loss of this fundamental spliceosome component has been reported to regulate flowering time in *Arabidopsis* by altering the expression patterns of several flowering related genes including *FLM* (Park et al., 2019). The observations that CDKG2 and CYCL1 control the AS of both *CDKG1* and *FLM* along the ambient temperature range, place this complex at the top of a signal transduction cascade translating environmental signals into developmental changes by regulating the AS of key regulatory genes in the temperature pathway.

Indeed, our data suggest a model whereby interplay between temperature and CDKs can modulate flowering time *via* AS of key floral regulators. We speculate that the flowering phenotype observed in *cdkg2* mutant lines may go beyond just a direct action on FLM considering that additional flowering genes are affected at the expression or AS levels (like *SVP*). A deeper understanding of the genetic interactions between CDKG related functions and the flowering time pathway could provide insights into the role of AS in regulating flowering and, particularly, the role it might play in temperature compensation.

However, whether temperature related differences in AS pertains to mRNA secondary structure modifications, as in the yeast model (Meyer et al., 2011) or to a sensor mediated signaling cascade, the molecular mechanisms ruling temperature dependent mRNA processing are yet to be fully elucidated.

The complexity and plasticity of the environmental sensing landscape in plants is only just emerging (Legris et al., 2016; Fujii et al., 2017; Casal and Qüesta, 2018; Dickinson et al., 2018; Wang et al., 2018; Han et al., 2019; Paik and Huq, 2019) and our results highlight the capacity of AS to bridge the interactions between environmental input pathways, specifically temperature, and central regulatory mechanisms, such as the cyclin dependent protein kinases, to control gene expression.

## Data Availability Statement

All datasets generated for this study are included in the article/**Supplementary Material**.

## Author Contributions

NC and CN conceived the project and designed research. NC, CN, MG, and DD performed research. NC and CN analyzed data. NC, CN, and JD wrote the paper. JD supervised the project and obtained funding.

## Funding

CN, DD, and JD were funded by the BBSRC (grant number BB/M009459/1). NC was funded by the VIPS Program of the Austrian Federal Ministry of Science and Research and the City of Vienna.

## Conflict of Interest

The authors declare that the research was conducted in the absence of any commercial or financial relationships that could be construed as a potential conflict of interest.
