# Supplementary material for: Thermo-Sensitive Alternative Splicing of FLOWERING LOCUS M Is Modulated by Cyclin-Dependent Kinase G2
Source: Front Plant Sci. 2020 Jan 22;10:1680. doi: 10.3389/fpls.2019.01680 (PMC6987439; doi:10.3389/fpls.2019.01680)
Supplement: Supplementary file 1 [file Table_1.docx]

Supplementary Material

**Supplementary Table 1.** List of the genes investigated by RT-PCR and/or RT-qPCR in Col-0, *cdkg1-1*, *cdkg2-1*, *cycl1-1* and the double *cdkg2-1*;*cycL1-1* double mutant lines.

| **Gene name** | **Gene ID** | **Gene name** | **Gene ID** | **Gene name** | **Gene ID** |
| --- | --- | --- | --- | --- | --- |
| *CDKG1* | AT5G63370 | *CDKG2* | AT5G63370 | *CYCL1* | AT2G26430 |
| *ATSRP30* | AT1G09140 | *ATSRP31* | AT3G61860 | *ATRSP31A* | AT2G46610 |
| *ATSRP34A* | AT3G49430 | *PTB1* | AT3G01150 | *ATSRP40* | AT4G25500 |
| *ATRS2Z32* | AT3G53500 | *ATRS2Z33* | AT2G37340 | *ATSCL30A* | AT3G13570 |
| *ATSRP34* | AT1G02840 | *ATSRP41* | AT5G52040 | *ATSCL33* | AT1G55310 |
| *ATRSZ22* | AT4G31580 | *U2AF65A* | AT4G36690 | *U2AF65B* | AT1G60900 |
| *ATSF1* | AT5G51300 | *CCA1* | AT2G46830 | *PRR9* | AT2G46790 |
| *TOC1* | AT5G61380 | *FCA* | AT4G16280 | *FPA* | AT2G43410 |
| *FLK* | AT3G04610 | *FLC* | AT5G10140 | *FLM* | AT1G77080 |
| *MAF2* | AT5G65050 | *SVP* | AT2G22540 | *TEM1* | AT1G25560 |
| *TEM2* | AT1G68840 |  |  |  |  |

**Supplementary Table 2. List of RT-PCR, RT-qPCR and cloning primers used.**

| **Primer name** | **Sequence** | **Application** | **Related publication** |
| --- | --- | --- | --- |
| FLMβ-δF | TGAATCCTCCGTCGCTGTTG | RT-PCR of *FLM* |  |
| FLMβ-δR | TCAAGCTTGCTTTGGACTGT | RT-PCR of *FLM* |  |
| MAF2-F | ATGGGTAGAAAAAAAGTCGAG | RT-PCR of *MAF2* | (Airoldi et al., 2015) |
| MAF2-R | CTTGAGCAGCGGAAGAGTCTCC | RT-PCR of *MAF2* | (Airoldi et al., 2015) |
| FLCin2-F | TTATGCATACCGCAATTTTCATAGC | RT-PCR of *FLC* | (Park et al., 2019) |
| FLCin2-R | ATTTAAGGTGGCTAATTAAGTAGTG | RT-PCR of *FLC* | (Park et al., 2019) |
| SVP1-F | CTGTAGCTCCAGCATGAAGGAA | RT-PCR of *SVP* | (Park et al., 2019) |
| SVP2-F | GAAAACTGTTCGACATGAAGGAAG | RT-PCR of *SVP* | (Park et al., 2019) |
| SVP-R | TGAAGTTCGCTGATCTCACTCATA | RT-PCR of *SVP* | (Park et al., 2019) |
| ATSF1-F | TTAATGCACCTTGGGCACCT | RT-PCR of *ATSF1* |  |
| ATSF1-F | TTCTCATGGCCTTTCTGCAA | RT-PCR of *ATSF1* |  |
| ATGRP7-F | CCGGTGATGTTGAGTATCGGT | RT-PCR of *ATGRP7* |  |
| ATGRP7-R | CCTCGTGACTGAGCCTCGTT | RT-PCR of *ATGRP7* |  |
| FLMe2-4F | CTTAGAGCCTTAGATCTTGAAG | qPCR of *FLM* | (Capovilla et al., 2017) |
| FLMe5-4R | CTTCAAGCTTGCTTTGGACTG | qPCR of *FLM* | (Capovilla et al., 2017) |
| FLMe1-3F | CCTCCGGTGACGAGATAGAAG | qPCR of *FLM* | (Capovilla et al., 2017) |
| FLMe4-3R | GAATTTTTTCTTCAAGATCGAG | qPCR of *FLM* | (Capovilla et al., 2017) |
| FLMe3-4F | GTTTTGAACTCGATCTTGAAG | qPCR of *FLM* | (Capovilla et al., 2017) |
| FLMi4R | GAGGGGAGAAAAATGTGTCG | qPCR of *FLM* | (Capovilla et al., 2017) |
| FLMi1F | GAGCTGTCATGACCTTCCCG | qPCR of *FLM* |  |
| FLMi1R | TGACGCTTTCACACATAACGTC | qPCR of *FLM* |  |
| FLMex1F | CCAAACGACGCAATGGTCTC | qPCR of *FLM* |  |
| FLMex1R | GCAGATACGACGACAACAGC | qPCR of *FLM* | (Sureshkumar et al., 2016) |
| FLMi1e2F | AGCTTTCTTTCACTTTGGTGC | RT-PCR of *FLM* |  |
| SVPqF | CAAGGACTTGACATTGAAGAGCTTCA | qPCR of *SVP* | (Posé et al., 2013) |
| SVPqR | CTGATCTCACTCATAATCTTGTCAC | qPCR of *SVP* | (Posé et al., 2013) |
| FLCqF | CTTGTGGGATCAAATGTCAAAAATGTG | qPCR of *FLC* | (Posé et al., 2013) |
| FLCqR | CATCTCAGCTTCTGCTCCCACATGATG | qPCR of *FLC* | (Posé et al., 2013) |
| TEM1qF | ATCCACTGGAAAGTCCGGTCTA | qPCR of *TEM1* | (Aguilar-Jaramillo et al., 2019) |
| TEM1qR | GAATAGCCTAACCACAGTCTGAACC | qPCR of *TEM1* | (Aguilar-Jaramillo et al., 2019) |
| TEM2qF | TGGTCCGAGAGAAAACCCG | qPCR of *TEM2* | (Aguilar-Jaramillo et al., 2019) |
| TEM2qR | TCAACTCCGAAAAGCCGAAC | qPCR of *TEM2* | (Aguilar-Jaramillo et al., 2019) |
| PP2A-F | TAACGTGGCCAAAATGATGC | Reference gene for qPCR | (Czechowski, 2005) |
| PP2A-R | GTTCTCCACAACCGCTTGGT | Reference gene for qPCR | (Czechowski, 2005) |
| SEFex1F | GGAAGAGATGTCGAACCGTCG | RT-PCR of *SEF* | (Gohring et al., 2014) |
| SEFex4R | GCTGCTTTCAAGTAAGTCGG | RT-PCR of *SEF* | (Gohring et al., 2014) |
| AtCDKG2 attB1 | GGGGACAAGTTTGTACAAAAAAGCAGGCTTGATGGCGGCTGGGAGGAATATAAG | Cloning |  |
| CDKG2-GFP-F | GGCGGTCTGTTTGGCGGAGGTGGTGGCGGCCGCATGGTGAGCAAGG | Cloning |  |
| GFP cter attB2 | GGGGACCACTTTGTACAAGAAAGCTGGGTTTTA CTT GTA CAG CTC GTC CAT GC | Cloning |  |


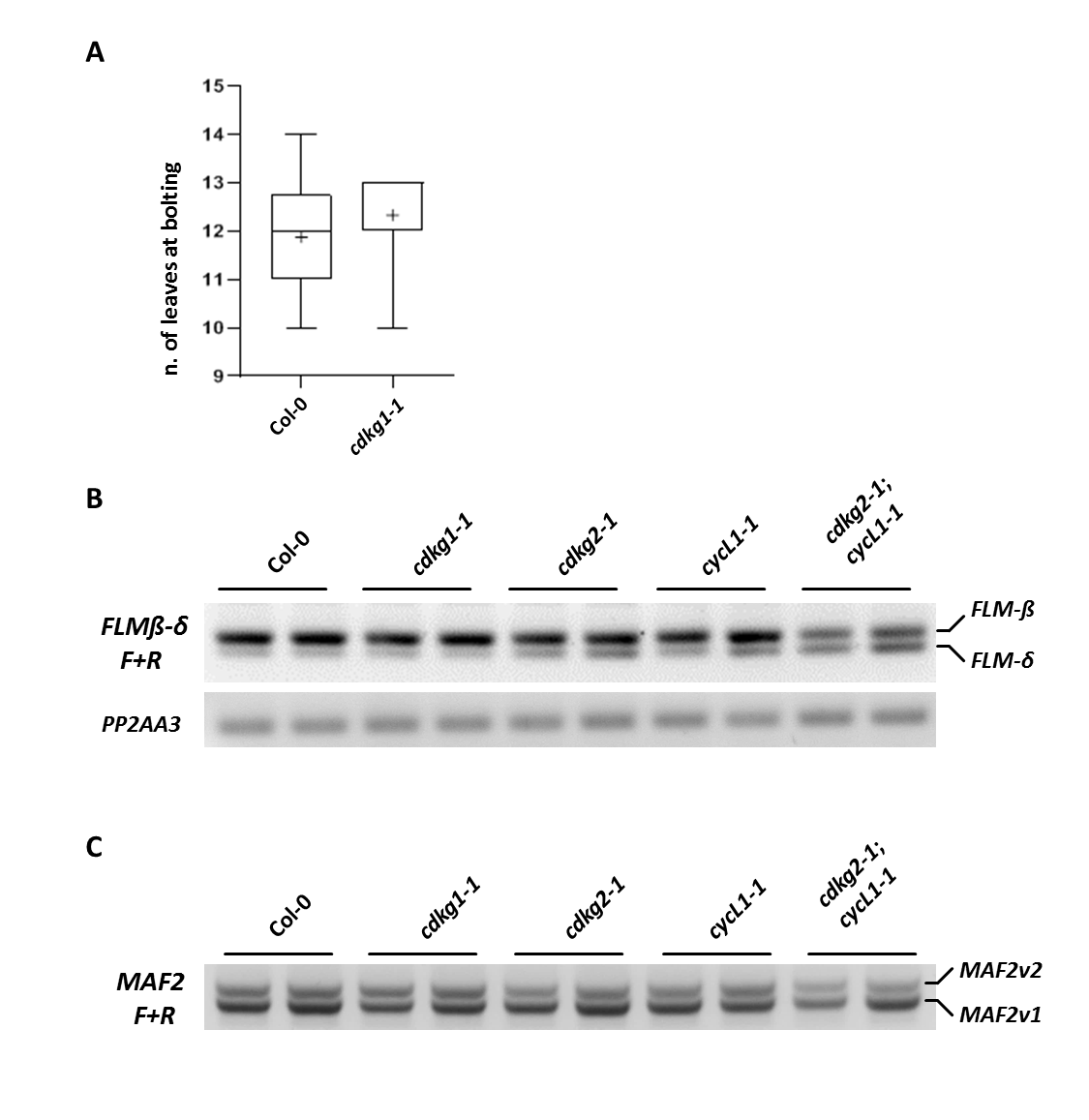


**Supplementary Figure 1. Flowering time and gel separation of RT-PCR products showing the relative abundance of the major splicing forms of *FLM* (*FLM-ß* and *FLM-δ*) and MAF2 at ambient temperature. (A)** Flowering time of Col-0 and *cdkg1-1* plants grown at 23°C under LD conditions quantified by counting the number of rosette leaves present at bolting (n ≥ 16). Boxes represent 2^nd^ and 3^rd^ quartiles, bars minimum to maximum values and crosses average of the groups. **(B)** Gel separation of RT-PCR products showing the relative abundance of the major splicing forms of *FLM* (*FLM-ß* and *FLM-δ*) in the different mutant backgrounds at 23°C (upper panel) and *PP2AA3* control (lower panel) in two independent samples as indicated. **(C)** Gel separation of RT-PCR products resulting from *MAF2* variant 1 and 2 (*MAF2v1* and *MAF2v*2) splicing in the different mutant backgrounds at 23°C in two independent samples as indicated.


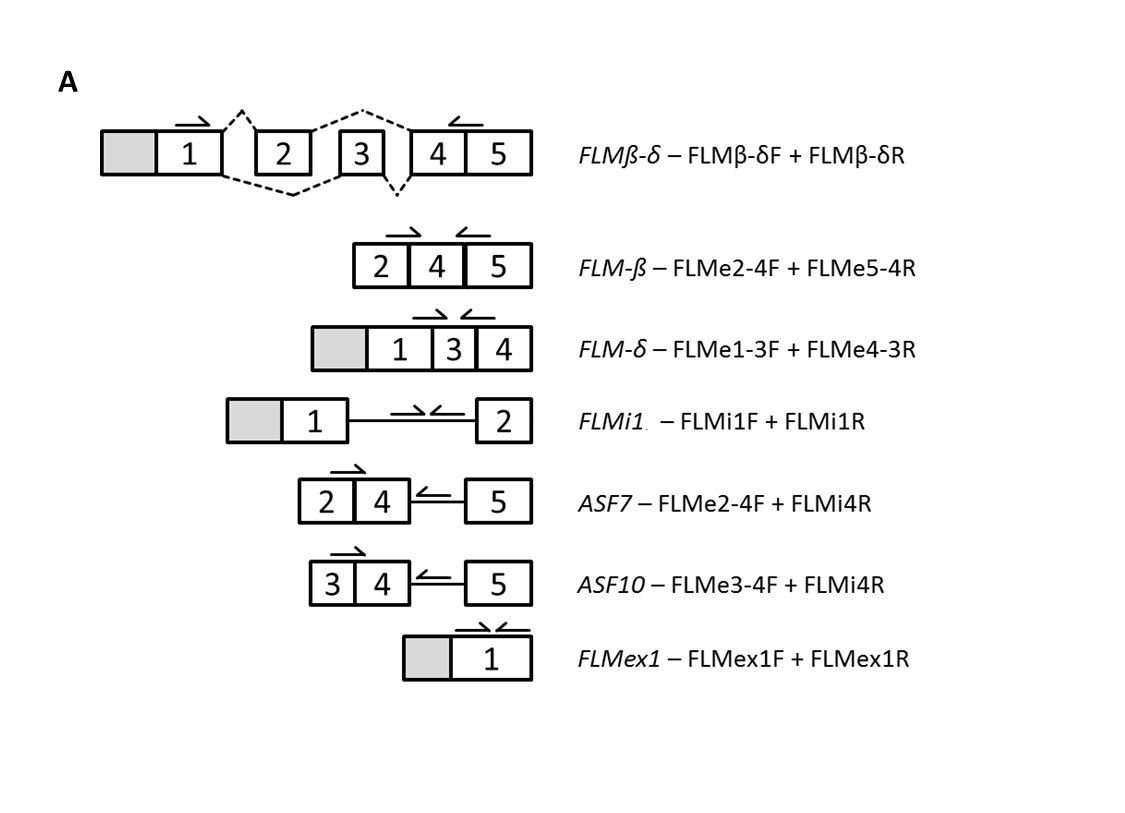


**Supplementary Figure 2. (A)** Schematic representation of the FLM gene structure, including exons (boxes) and introns (lines). White boxes correspond to coding exons, grey boxes correspond to non‐coding exon sequences (UTRs). Dotted lines represent alternative splicing (AS) events. The primers used to specifically amplify each isoform are indicated (primer sequences can be found in Table S2).


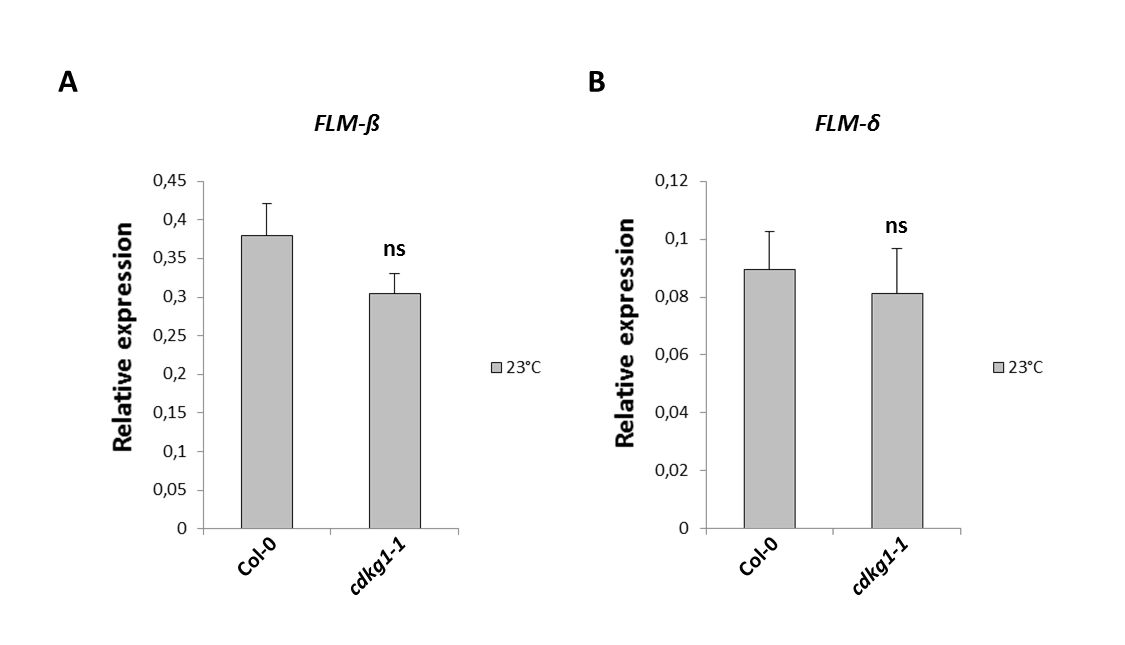


**Supplementary Figure 3. (A)** and **(B)** Relative expression levels of *FLM-ß* (B) and *FLM-δ* (C) mRNA as quantified by RT-qPCR in wild type and *cdkg1-1* mutant grown at 23°C under LD conditions. Student’s t-test comparing c*dkg1-1* to Col-0 at 23°C, n = 3, ns - not significant.


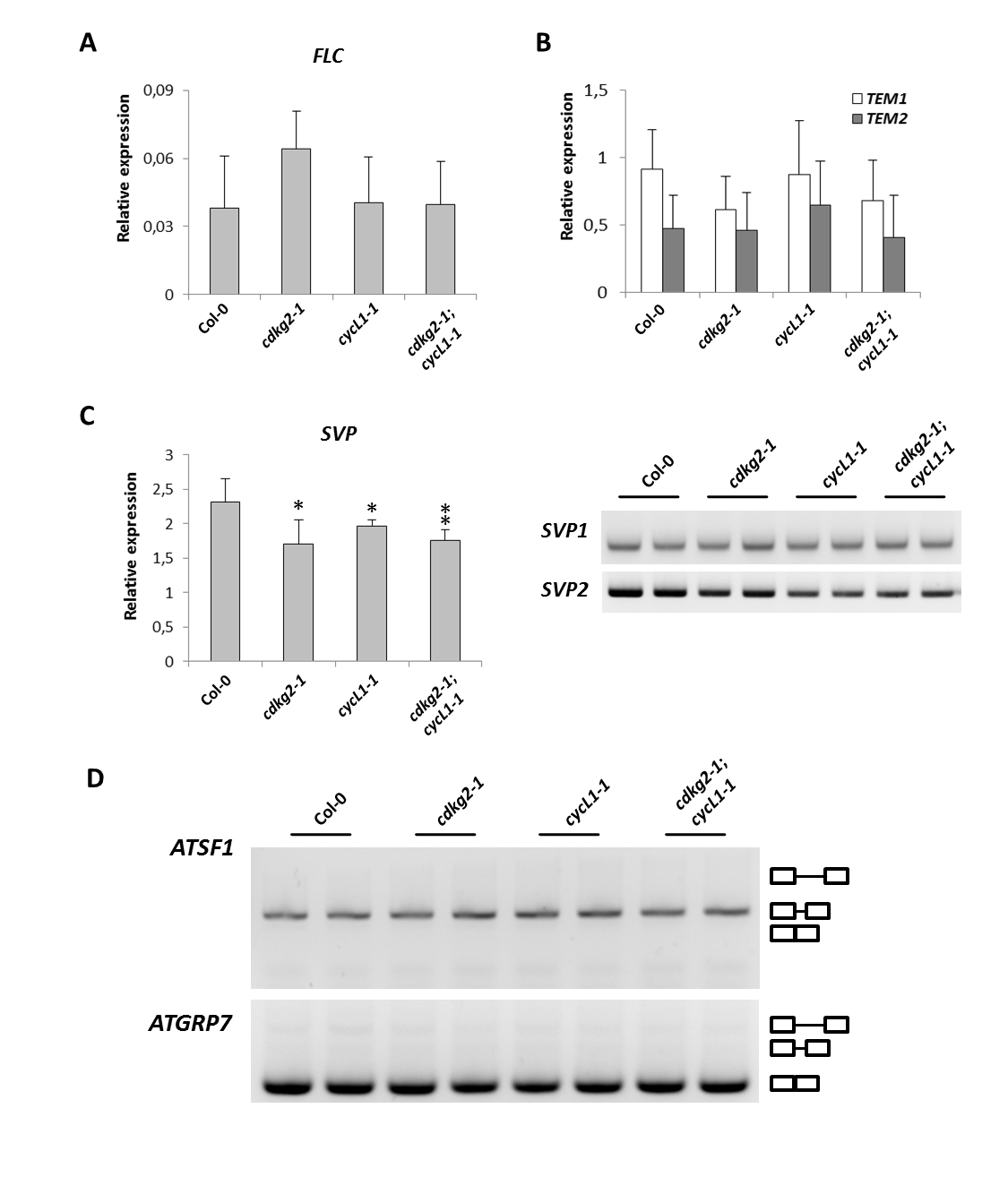


**Supplementary Figure 4. RT-qPCR and gel separation of RT-PCR products of flowering regulatory genes at ambient temperature. (A,B)** Relative expression levels of *FLC* **(A)** and *TEM1, TEM2* **(B)** mRNA as quantified by RT-qPCR in the different lines (n ≥ 3). **(C)** Relative expression levels of total *SVP* mRNA as quantified by RT-qPCR (left panel, n ≥ 5) and gel separation of RT-PCR products showing the relative abundance of two splicing forms of *SVP* (*SVP1* and *SVP2*) in the different mutant backgrounds in two independent samples (right panels). **(D)** Gel separation of RT-PCR products resulting from *ATSF1* alternative splicing (upper panel) and *ATGRP7* (lower panel) in the different mutant backgrounds in two independent samples. The schematic gene structure of alternatively spliced transcripts are depicted according to the predicted bp size: boxes and lines indicate exons and introns respectively.

Student’s t-test comparing c*dkg2-1*, *cycL1-1* or *cdkg2-1*;*cycL1-1* to Col-0 grown at 23°C under LD conditions,, ** p<0.01 and *p<0.05.


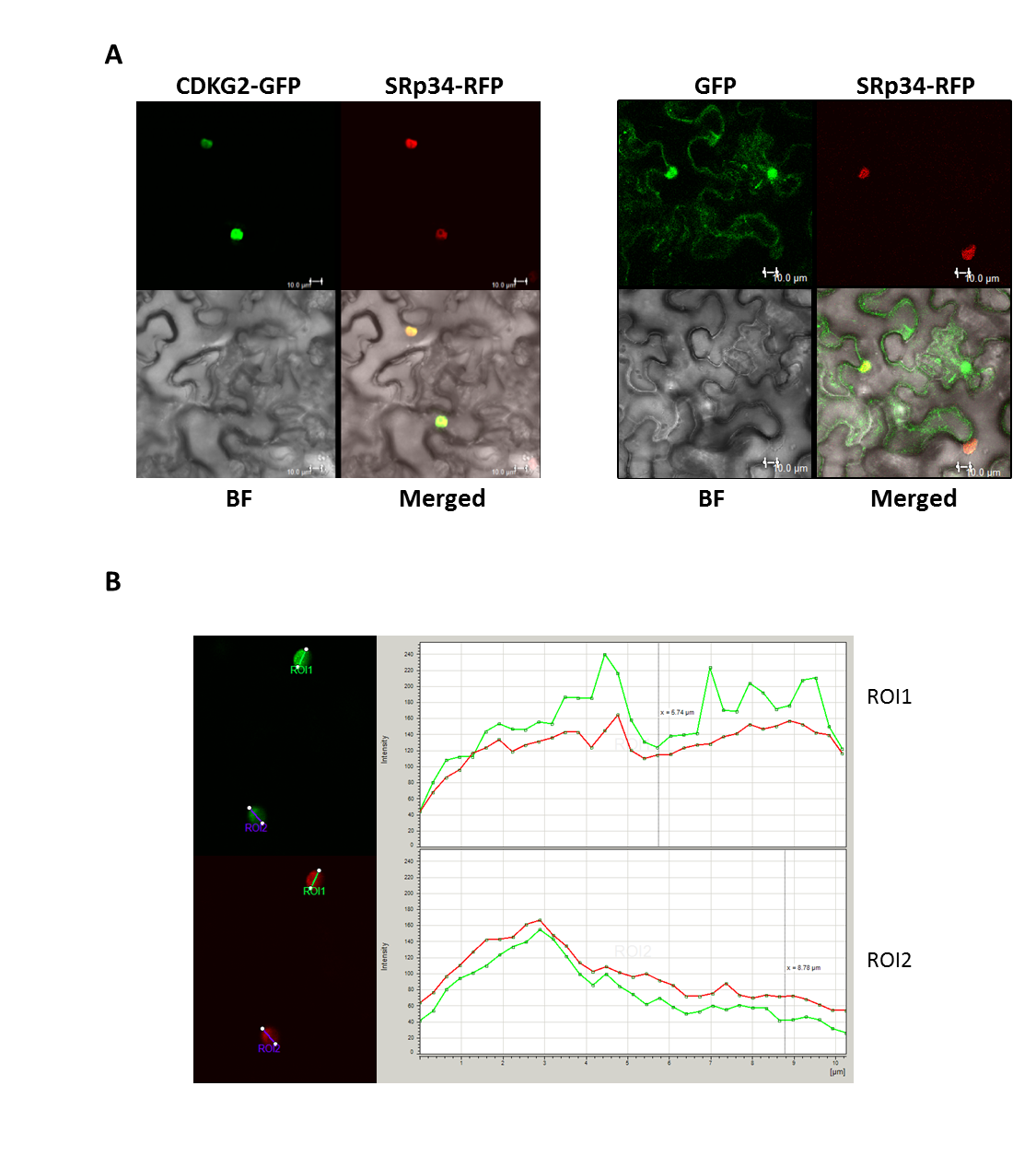


**Supplementary Figure 5. Analysis of CDKG2-GFP protein expression.** **(A)** CDKG2-GFP protein co-localises with the spliceosome component RSp34-RFP in *N. benthamiana* leaves. The green channel (GFP) shows the localisation of CDKG2-GFP protein, the red (RFP) channel the localization of the RSp34-RFP protein, BF is a bright field image of the leaf region and Merged an overlay of the three channels. Localization of the GFP control protein is shown on the right hand panel. **(B)** Fluorescence intensity plot showing co-localisation of the green and red signals across the dotted lines as indicated, for two nuclei (ROI1 and ROI2).


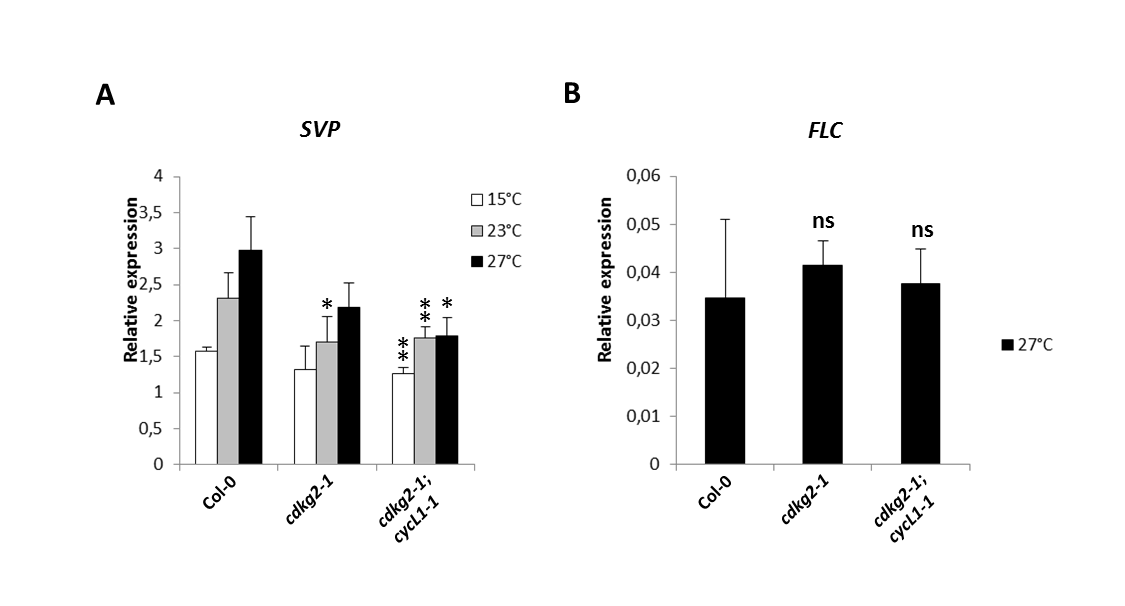


**Supplementary Figure 6. Analysis of *SVP* and *FLC* expression. (A)** Quantification of *SVP* total mRNA levels estimated by RT-qPCR in Col-0 and in mutant seedlings. **(B)** Quantification of *FLC* total mRNA levels estimated by RT-qPCR in Col-0 and in mutant seedlings. 2 week old seedlings were grown at the indicated temperatures under long day conditions before mRNA extraction. Student’s t-test comparing *cdkg2-1* or *cdkg2-1*;*cycL1-1* to Col-0 at the respective temperature, n = 3, **p<0.01, *p<0.05, ns - not significant.


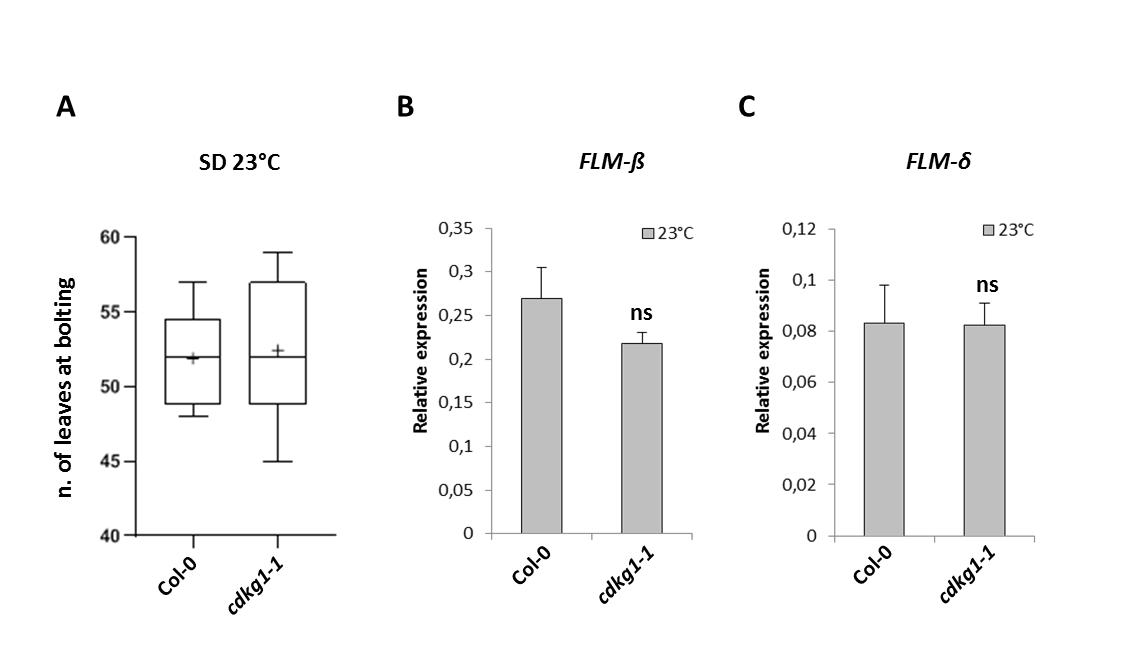


**Supplementary Figure 7. Flowering time of Col-0 and *cdkg1-1* in Short Day. (A)** Flowering time of Col-0 and *cdkg1-1* plants grown at 23°C under SD conditions quantified by counting the number of rosette leaves present at bolting (n ≥ 10). Boxes represent 2^nd^ and 3^rd^ quartiles, bars minimum to maximum values and crosses average of the groups. **(B)** and **(C)** Relative expression levels of *FLM-ß* (B) and *FLM-δ* (C) mRNA as quantified by RT-qPCR in the different lines grown at 23°C under SD conditions. Student’s t-test comparing c*dkg1-1* to Col-0 at 23°C, n = 3, ns - not significant.


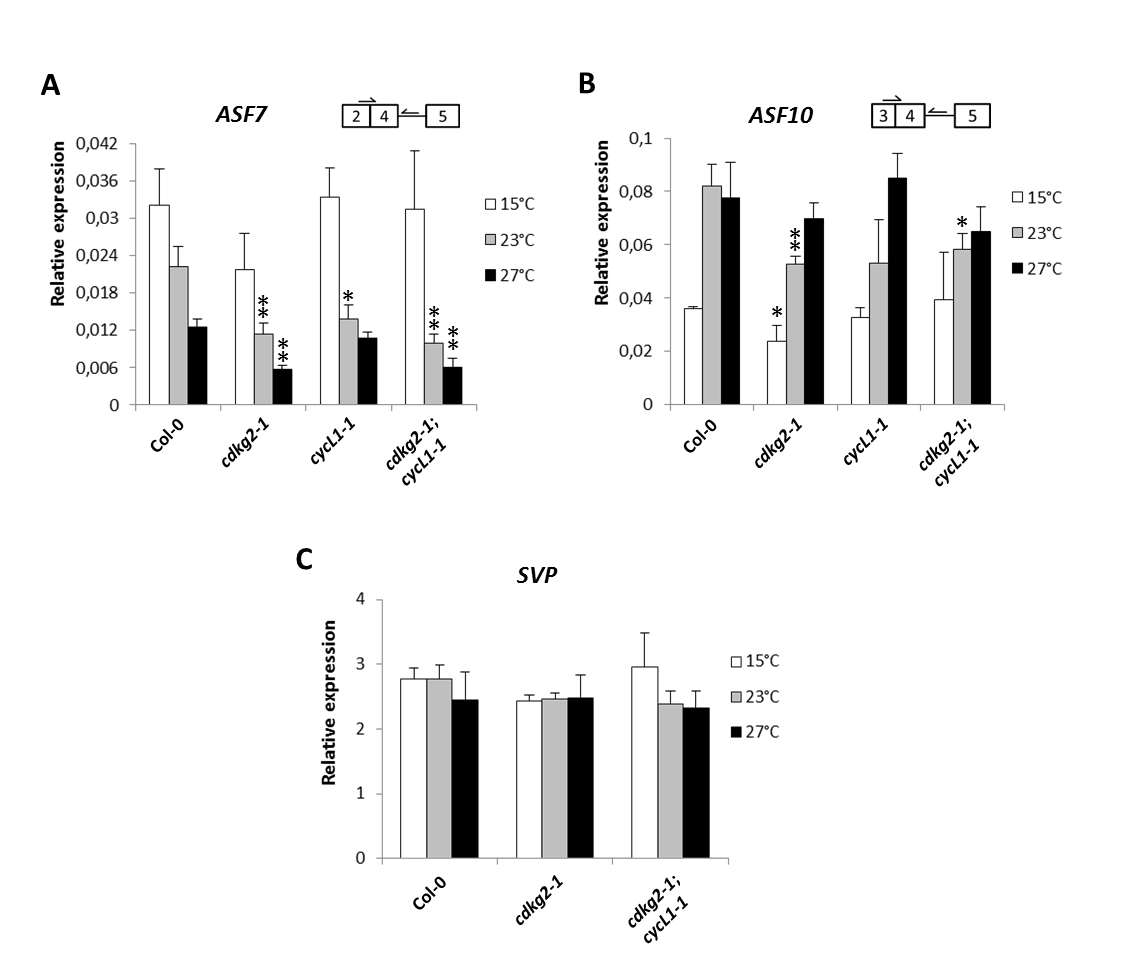


**Supplementary Figure 8. The CDKG2 and CYCL1 regulate the abundance of *ASF7* and *ASF10* transcripts but not *SVP* under short day conditions.** Relative expression levels of *ASF7* **(A)**, *ASF10* **(B)** and *SVP* **(C)** in 2 week old seedlings grown at the indicated temperatures under short day conditions before mRNA extraction. Student’s t-test comparing *cdkg2-1*, *cycL1-1* or *cdkg2-1*;*cycL1-1* to Col-0 at the respective temperature, n = 3, ** p<0.01, *p<0.05, ns – not significant.


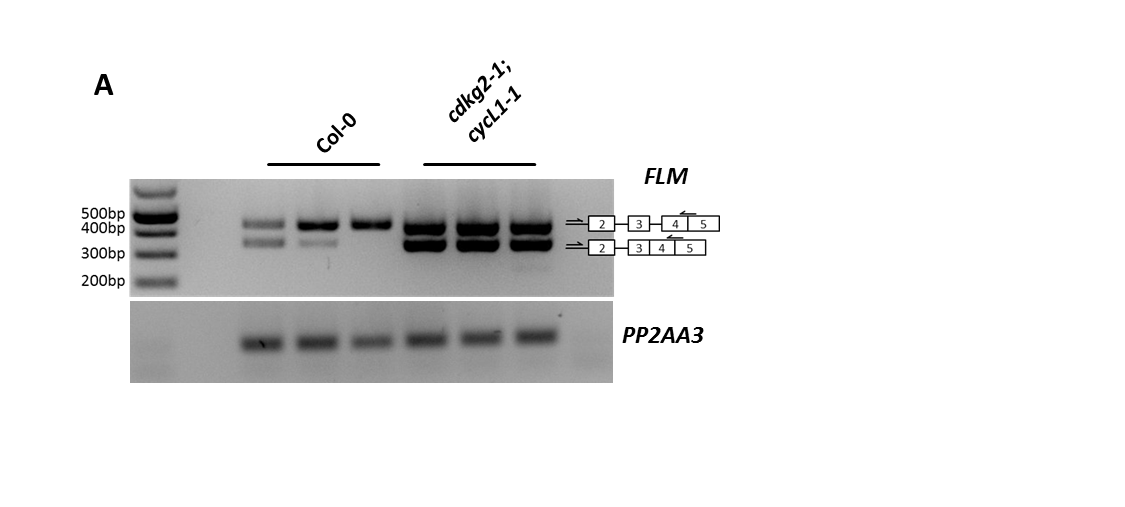


**Supplementary Figure 9. intron 1 retention *FLM* mRNAs. (A)** *FLM* splicing pattern after gel separation of RT-PCR product using primers FLMi1e2F+FLMe5-4R (upper panel) and *PP2AA3* control (lower panel) in three independent samples for Col-0 and *cdkg2-1;cycL1-1* seedlings grown at 27°C under long day conditions. Putative transcripts are depicted on the right side of the image according to base pair length.

Aguilar-Jaramillo, A.E., Marín-González, E., Matías-Hernández, L., Osnato, M., Pelaz, S., Suárez-López, P., 2019. TEMPRANILLO is a direct repressor of the micro RNA miR172. Plant J tpj.14455. https://doi.org/10.1111/tpj.14455

Airoldi, C.A., McKay, M., Davies, B., 2015. MAF2 Is Regulated by Temperature-Dependent Splicing and Represses Flowering at Low Temperatures in Parallel with FLM. PLOS ONE 10, e0126516. https://doi.org/10.1371/journal.pone.0126516

Capovilla, G., Symeonidi, E., Wu, R., Schmid, M., 2017. Contribution of major FLM isoforms to temperature-dependent flowering in Arabidopsis thaliana. Journal of Experimental Botany 68, 5117–5127. https://doi.org/10.1093/jxb/erx328

Czechowski, T., 2005. Genome-Wide Identification and Testing of Superior Reference Genes for Transcript Normalization in Arabidopsis. PLANT PHYSIOLOGY 139, 5–17. https://doi.org/10.1104/pp.105.063743

Gohring, J., Jacak, J., Barta, A., 2014. Imaging of Endogenous Messenger RNA Splice Variants in Living Cells Reveals Nuclear Retention of Transcripts Inaccessible to Nonsense-Mediated Decay in Arabidopsis. The Plant Cell 26, 754–764. https://doi.org/10.1105/tpc.113.118075

Park, H.-Y., Lee, H.T., Lee, J.H., Kim, J.-K., 2019. Arabidopsis U2AF65 Regulates Flowering Time and the Growth of Pollen Tubes. Front. Plant Sci. 10, 569. https://doi.org/10.3389/fpls.2019.00569

Posé, D., Verhage, L., Ott, F., Yant, L., Mathieu, J., Angenent, G.C., Immink, R.G.H., Schmid, M., 2013. Temperature-dependent regulation of flowering by antagonistic FLM variants. Nature 503, 414–417. https://doi.org/10.1038/nature12633

Sureshkumar, S., Dent, C., Seleznev, A., Tasset, C., Balasubramanian, S., 2016. Nonsense-mediated mRNA decay modulates FLM-dependent thermosensory flowering response in Arabidopsis. Nature Plants 2, 16055. https://doi.org/10.1038/nplants.2016.55
